# An Optimised Di-Boronate-ChemMatrix Affinity Chromatography to Trap Deoxyfructosylated Peptides as Biomarkers of Glycation

**DOI:** 10.3390/molecules25030755

**Published:** 2020-02-10

**Authors:** Monika Kijewska, Francesca Nuti, Magdalena Wierzbicka, Mateusz Waliczek, Patrycja Ledwoń, Agnieszka Staśkiewicz, Feliciana Real-Fernandez, Giuseppina Sabatino, Paolo Rovero, Piotr Stefanowicz, Zbigniew Szewczuk, Anna Maria Papini

**Affiliations:** 1 Faculty of Chemistry, University of Wrocław, ul. F. Joliot-Curie 14, 50-383 Wrocław, Poland; magdalena.wierzbicka@chem.uni.wroc.pl (M.W.); mateusz.waliczek@chem.uni.wroc.pl (M.W.); patrycja.ledwon@pwr.edu.pl (P.L.); agnieszka.staskiewicz@pwr.edu.pl (A.S.); piotr.stefanowicz@chem.uni.wroc.pl (P.S.); zbigniew.szewczuk@chem.uni.wroc.pl (Z.S.); 2 Laboratory of Peptide and Protein Chemistry and Biology, Department of Chemistry “Ugo Schiff”, University of Florence, Via della Lastruccia 13, 50019 Sesto Fiorentino, Italy; francesca.nuti@unifi.it (F.N.); feliciana.realfernandez@unifi.it (F.R.-F.); giuseppina.sabatino@cnr.it (G.S.); 3 Laboratory of Peptide and Protein Chemistry and Biology, Department of Neurosciences, Psychology, Drug Research and Child Health—Section of Pharmaceutical Sciences and Nutraceutics, University of Florence, Via Ugo Schiff 6, 50019 Sesto Fiorentino, Italy; paolo.rovero@unifi.it; 4 CNR-IC Istituto di Cristallografia, Via Paolo Gaifami 18, 95126 Catania, Italy; 5 PeptLab@UCP and Laboratory of Chemical Biology EA4505, CY Cergy Paris University, 5 Mail Gay-Lussac, 95031 Cergy-Pontoise, France

**Keywords:** phenylboronate, affinity chromatography, ChemMatrix^®^ Rink resin, N^ε^-deoxyfructosyl-lysine, LC-MS analysis

## Abstract

We report herein a novel ChemMatrix^®^ Rink resin functionalised with two phenylboronate (PhB) moieties linked on the *N*-α and *N*-ε amino functions of a lysine residue to specifically capture deoxyfructosylated peptides, compared to differently glycosylated peptides in complex mixtures. The new PhB-Lys(PhB)-ChemMatrix^®^ Rink resin allows for exploitation of the previously demonstrated ability of *cis* diols to form phenylboronic esters. The optimised capturing and cleavage procedure from the novel functionalised resin showed that only the peptides containing deoxyfructosyl-lysine moieties can be efficiently and specifically detected by HR-MS and MS/MS experiments. We also investigated the high-selective affinity to deoxyfructosylated peptides in an ad hoc mixture containing unique synthetic non-modified peptides and in the hydrolysates of human and bovine serum albumin as complex peptide mixtures. We demonstrated that the deoxyfructopyranosyl moiety on lysine residues is crucial in the capturing reaction. Therefore, the novel specifically-designed PhB-Lys(PhB)-ChemMatrix^®^ Rink resin, which has the highest affinity to deoxyfructosylated peptides, is a candidate to quantitatively separate early glycation peptides from complex mixtures to investigate their role in diabetes complications in the clinics.

## 1. Introduction

In high-glucose concentration, typical of different diabetes conditions, the reducing sugar reacts with free amino functions, such as the *N^ε^*-side chain of lysine residues in proteins, to form, by a nonenzymatic reaction, the so-called Schiff bases. Those imines are not stable and after the Amadori rearrangement, the more stable 1-deoxyfructosyl moiety is formed, leading to *N^ε^*-deoxyfructosyl-lysine residues in proteins, usually termed early glycation adducts. Many different advanced glycation end products (AGEs) are then formed in vivo, such as oxoaldehydes and α,β-diketones, by subsequent dehydration, condensation, fragmentation, oxidation, and cyclization reactions of the *N^ε^*-deoxyfructosyl-lysines. Reactive dicarbonyls, glyoxal, methylglyoxal, and 3-deoxyglucosone are potent glycation agents, highly reactive more than the glucose itself and thus originate glycation chain reactions on more and more protein sites [1,2,3]. AGEs and their concentration levels are considered, in the clinics, relevant for diagnosis of diabetes complications and proteomics technologies are proposed for their characterisation [4,5,6,7]. However the possibility to trap early glycation adducts (i.e., deoxyfructosylated proteins) and the corresponding peptides by efficient mass spectrometry technology is a challenge to anticipate AGEs formation. In fact, gradual accumulation of proteins aberrantly modified with deoxyfructosyl-lysines, earlier, and AGEs, later, in the blood vessel walls cause pathological effects responsible for diabetes-related micro- and macro-vascular diseases [8]. Analysis of such aberrantly modified peptides is extremely difficult due to their low abundance in biological fluids and complexity of the sample. Therefore, efficient methods for isolation, enrichment, and recognition of glycated proteins are of current research interest.

Hereof, boronate affinity materials (BAMs) have gained increasing attention in recent years [9], because of the specificity of the reaction of boronic acid that has been demonstrated efficient for the separation of a wide variety of *cis* diol-containing compounds, including catechols, nucleosides, nucleotides, nucleic acids, carbohydrates [10], glycoproteins [6,7,11], and enzymes [12]. In particular, boronic acid forms with *cis* diols esters (in aqueous solution under basic conditions) that can be easily hydrolysed under acidic conditions. This reversible reaction is useful for selective detection of *cis* diol-containing compounds. Therefore, BAMs are commonly used for immobilisation, detection, and separation of glycoproteins [9]. To date, many ligands bearing the boronic acid moiety immobilised on monoliths [13], magnetic particles [14], mesoporous silica [15], polymer nanoparticles [16], and gold nanoparticles [17] have been used for enrichment of *cis* diol-containing compounds. The first commercially available product, based on *m*-aminophenylboronic acid (*m*-APBA) immobilised on an agarose gel, was proposed for capturing immunoglobulins, glycosylated [18], but also glycated peptides (involved in diabetes) [6,19,20]. However the *m*-APBA support demonstrated some undesirable and non-specific interactions with peptides containing tyrosine, threonine, and/or serine residues that can dramatically affect the efficiency of the method [19]. Despite these non-specific interactions, the proposed method allowed enrichment of the mixture with glycated peptides [19]. In particular, incorporation of electron-withdrawing groups into the phenylboronic acid moiety [21], but also heterocyclic analogues of boronic acids [22], required a low-binding pH. For example, functionalisation of monolithic capillary with BSPBA (e.g., the 3-butenylsulfonyl-phenylboronic acid) allowed capturing nucleosides and glycoproteins at neutral pH [21]. Magnetic nanoparticles with dendrimer-boronate moieties enhanced binding of glycoproteins, demonstrating the effectiveness of a multivalent binding strategy [16]. 

We previously reported on the ability of *cis* diols to form boronic, but also phenylboronic esters, demonstrating that boronate and phenylboronate complexes of peptides containing the deoxyfructosyl-lysine moiety can be efficiently and specifically detected by HR-MS and MS/MS experiments [11,23]. In particular, stabilisation of the deoxyfructosyl moiety by boronate and phenylboronate derivatives, stabilised in MS/MS conditions, makes their fragmentation pattern simpler and; therefore, easier to elucidate. Moreover, we demonstrated that derivatisation of phenylboronic acid as quaternary ammonium salts (QAS) improves the efficiency of mass spectrometry ionisation. Therefore, these derivatives allowing to discriminate glycosylated peptides *versus* deoxyfructosylated peptides (useful tools as biomarkers of early glycation), make reliable mass spectra obtained by the technique proposed herein [23].

In the current study, we selected the ChemMatrix^®^ Rink resin (CMRR) to increase selectivity of detection of deoxyfructosylated peptides as an alternative to the previously proposed *m*-aminophenylboronic acid (*m*-APBA) immobilised on the agarose gel [6]. In particular, the advantages of the ChemMatrix^®^ Rink resin are: compatibility with a large variety of solvents, including water; high chemical stability; optimal swelling properties; and the possibility to build on it any type of peptide sequence. Moreover, the ChemMatrix^®^ resin (initially designed for solid-phase synthesis of difficult peptide sequences, including long and hydrophobic ones) [24,25], was recently used for peptide-affinity chromatography based on combinatorial strategies for protein purification [26]. Last, but not least, derivatisation of the CMRR is relatively simple following the optimised protocols set up for peptide synthesis [26]. 

**Scheme 1:**
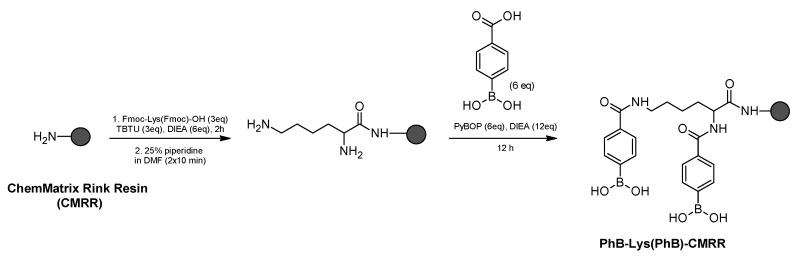
Synthesis of the new functionalised PhB-Lys(PhB)-ChemMatrix^®^ Rink resin.

Therefore, CMRR has been selected to design a novel stationary phase for affinity chromatography of deoxyfructosylated peptides. In particular, we propose herein a simple synthetic protocol using the low-cost Fmoc-Lys(Fmoc)-OH as starting material to act as a linker between the CMRR and two phenylboronate moieties (Scheme 1). In particular, we developed the PhB-Lys(PhB)-CMRR as a capturing support for reversible binding of deoxyfructosylated peptides for further mass spectrometry identification. According to the literature, there is no evidence that the lysine moiety with two phenylboronate moieties linked to CMRR has been reported and applied for a specific affinity chromatography technique. Therefore, we examined the peptide-capturing properties of the novel functionalised resin PhB-Lys(PhB)-CMRR as a function of time to determine its efficiency. We confirmed its stability in different solvents and we developed analytical methods to perform its quality control, including loading and homogeneity of PhB-Lys(PhB)-NH_2_ once cleaved from the resin. A synthetic protocol for the preparation of the novel functionalised resin PhB-Lys(PhB)-CMRR was optimised. We proved that the introduction of two phenylboronate moieties attached to the lysine linker has a significant impact on binding the deoxyfructosylated peptides. We showed that the PhB-Lys(PhB)-CMRR is recyclable having demonstrated the same ability to separate deoxyfructosylated peptides in solution after several cycles of capturing. Moreover, the resin was tested to demonstrate that deoxyfructosylated peptides could be captured specifically compared to other glycopeptides modified with different glycosyl moieties. In fact, its high selectivity and affinity for the deoxyfructosylated peptides were investigated using an ad hoc mixture containing unique synthetic glycopeptides and non-modified peptides, and complex peptide mixtures obtained from hydrolysis of human and bovine serum albumin (HSA and BSA).

## 2. Materials and Methods

### 2.1. Synthesis of the PhB-Lys(PhB)-ChemMatrix^®^ Rink Resin 

The ChemMatrix^®^ Rink resin (loading 0.4–0.6 mmol/g) was linked to Fmoc-Lys(Fmoc)-OH (3 eq) by 2-(1*H*-Benzotriazole-1-yl)-1,1,3,3-tetramethylaminium tetrafluoroborate (TBTU, 3 eq) in the presence of *N,N*-Diisopropylethylamine (DIEA) (6 eq) in 2 h. The reaction was monitored by ninhydrin test. After removal of Fmoc-protecting groups from the *N*-terminus and *ε*-amino function of lysine using 25% piperidine in DMF, the 4-carboxyphenylboronic acid (PhB-OH, 6 eq) was linked by (Benzotriazol-1-yl-oxy)tripyrrolidinophosphonium hexafluorophosphate (PyBOP) (6 eq) in 12 h in the presence of DIEA (12 eq). Then the functionalised resin was dried under *vacuum* for three days at room temperature. PhB-Lys(PhB)-NH_2_ was cleaved in 2 h from the resin using a mixture of TFA/H_2_O/TIS 95:2.5:2.5 (*v*:*v*:*v*). The solution was evaporated under a gentle stream of nitrogen and then lyophilised and used to evaluate final loading (details are reported in Supporting Information—SI). 

### 2.2. General Capturing Protocol of Model Deoxyfructosylated Peptide ***1***

PhB-Lys(PhB)-CMRR (5 mg) were swelled in a syringe for 30 min in ammonium bicarbonate buffer (50 mM) at pH 8 in H_2_O/MeCN 1:1 (*v*/*v*). Equimolar amount of deoxyfructosylated peptide H-K(Dabcyl)AK(1-DeoxyFru)AF-NH_2_ (**1**) was dissolved in the same buffer (1 mL), and added to the resin and mixed for 1 h. Then the solution was filtered off and the resin was washed three times with the buffer solution to remove the possible unreacted deoxyfructosylated peptide. All the fractions were collected and lyophilised. Then, the cleavage mixture containing 0.1% HCOOH in H_2_O/MeCN 1:1 (*v*/*v*) was added and the syringe was stirred for 1h. The solution was filtered off and the resin was washed four times with the cleavage mixture. All the cleavage fractions were collected, lyophilised, and analysed by LC-MS, LC-MS/MS, and UV-VIS (Scheme S1, see SI).

To regenerate the functionalised resin, it was washed with the same ammonium bicarbonate buffer (3 × 1 min) and then with DCM (3 × 1 min), THF (3 × 1 min), Et_2_O (3 × 1 min), and dried under *vacuum*. 

### 2.3. Capturing Conditions of Peptides ***2***–***8***

The general procedure optimised for peptide **1** was applied separately to the synthetic peptides **2**–**8** using equimolar concentrations of all the peptides (1.4 µmol/mL). The capturing reaction time was extended to 2 h. The remaining steps of the procedure were applied without changes. All the cleavage fractions were collected, lyophilised, and analysed by LC-UV-MS.

### 2.4. Capturing Conditions of Peptide ***2*** in the Mixture of Peptides ***7*** and ***9***–***17***

Three ad hoc aqueous solutions containing the equimolar concentrations of non-glycosylated peptide sequences (1.4 µmol/mL), including the deoxyfructosylated peptide **2**, at the same concentration (1.4 µmol/mL), or 5% *w*/*w* (10.5 nmol/mL), or 2% *w*/*w* (4.2 nmol/mL), were prepared and used following the general capturing protocol described above, extending the time to 2 h. The remaining steps of the protocol were applied without changes. All the fractions (uncaught and released from PhB-Lys(PhB)-ChemMatrix^@^ Rink resin) were collected, lyophilised, and analysed by LC-UV-MS.

### 2.5. Capturing Conditions of Peptide ***2*** and ***18*** in HSA or BSA Hydrolysate

Human Serum Albumin (HSA, BioLabs) was hydrolysed according to the procedure reported in SI. The deoxyfructosylated peptide **2** was added to the HSA hydrolysate at the same concentration used for the non-glycosylated peptides (1.4 μmol/mL), or 5% *w*/*w* (10.5 nmol/mL), or 2% *w*/*w* (4.2 nmol/mL), and applied in the general capturing protocol, also extending the time reaction to 2 h. The remaining steps were followed without any changes. All the cleavage fractions were collected, lyophilised, and analysed by LC-MS, LC-UV-MS, or LC-MS/MS. The LC-MS/MS data were analysed using PEAKS search engine.

The general protocol described above was applied to a mixture of the commercially available Bovine Serum Albumin hydrolysate (500 pmol, BSA, BioLabs) including peptide **18** (300 pmol), extending the time reaction to 2 h. The remaining steps were followed without any changes. All the cleavage fractions were collected, lyophilised, and analysed by LC-MS/MS. Obtained LC-MS/MS data were analysed using PEAKS search engine.

### 2.6. Bioinformatic Analysis with PEAKS

LC-MS/MS analyses of the samples were performed on a SHIMADZU IT-TOF instrument using automatic fragmentation mode. The spectra were analysed by a bioinformatic approach using the LabSolution software to convert LC data file to mzXML format. Data were analysed using PEAKS search engine. For this purpose, we downloaded a FASTA file from the UniProt database. The following search parameters were set: precursor mass error tolerance ±0.1 Da; fragment ion tolerance ±0.2 Da; CID fragmentation, semi-specific digestion mode by trypsin, assuming up to three missed cleavages per peptide. Moreover, carbamidomethylation, as a fixed post-translational modification (PTM), was chosen. Deamidation, dehydration, oxidation of methionine, and hexose modifications were set as variable PTMs and three maximum variable PTMs per peptide were allowed. A false discovery rate (FDR) < 2% at the spectra level was used to filter search results. Additionally, the presence of at least two unique peptides was set for the identification of the corresponding protein. The obtained results, especially the statistical data, were reported into the Supporting Information.

## 3. Results and Discussion

### 3.1. Development of a Specific ChemMatrix^®^ Rink Resin Functionalised to Selectively Capture Deoxyfructosylated Peptides 

We report herein a novel stationary phase designed and optimised for affinity chromatography technology, to selectively capture deoxyfructosylated peptides, as we demonstrated on different samples. We selected the ChemMatrix^®^ Rink resin (CMRR) as an ideal solid support for its well-described intrinsic properties [24,25]. Fmoc-Lys(Fmoc)-OH was used as a low-cost, commercially-available building block to introduce a linker between CMRR and two phenylboronate moieties (PhB). The proposed novel capturing support (e.g., PhB-Lys(PhB)-CMRR) was designed to carry two PhB (linked to the *N*-α and *N*-ε amino functions of the lysine residue respectively), to significantly improve trapping of deoxyfructosylated peptides. The CMRR is fully compatible with a solid-phase synthetic protocol to develop a collection of different phenylboronate derivatives. Therefore, the synthetic procedure (Scheme 1) initially binds Fmoc-Lys(Fmoc)-OH to CMRR using 2-(1*H*-Benzotriazole-1-yl)-1,1,3,3-tetramethylaminium tetrafluoroborate (TBTU) as a coupling reagent and, after removing the two Fmoc-protecting groups in a piperidine solution, the 4-carboxyphenylboronic acid (PhB-OH) was reacted with the two lysine-amino functions by PyBOP. We previously reported an efficient strategy to link PhB-OH to a lysine side chain without any protecting group on hydroxyl functions [23]. 

In contrast to the *m*-aminophenylboronate agarose support previously proposed [6], successful functionalisation and loading of the PhB-Lys(PhB)-ChemMatrix^®^ Rink resin can be easily and efficiently monitored by quantitative LC-ESI-MS determination of the acidic hydrolysis product PhB-Lys(PhB)-NH_2_. In fact, the HPLC (Figure S1) and ESI-MS spectrum profiles (Figure S2) of the PhB-Lys(PhB)-NH_2_ crude show an intensive signal corresponding to the *m*/*z* value and the characteristic isotopic pattern consistent with the hydrolysis product corresponding to the molecular formula C_20_H_25_N_3_O_7_B_2_Na (Figure S3).

A calibration curve was set up with the commercially available 4-carboxyphenylboronic acid (purity > 99%) as a standard (Figure S4). In particular the crude PhB-Lys(PhB)-NH_2_ (Figure S5) at 236 nm (not interfering with amide bond absorptions) [27] allows calculation of the loading of PhB-Lys(PhB)-ChemMatrix^®^ Rink resin, after cleavage of PhB-Lys(PhB)-NH_2_ from an exactly weighted quantity of resin, lyophilisation, and final UV analysis (Table 1).

We demonstrated that the novel PhB-Lys(PhB)-ChemMatrix^®^ Rink resin is stable to capture reaction conditions and the resin loading is constant. In fact, after incubation of the functionalised resin in different solvents, accurate ESI-MS analysis of the lyophilised solutions did not display any signal corresponding to the possible cleaved linker PhB-Lys(PhB)-NH_2_.

We used, as first model the simple synthetic pentapeptide visible sequence, H-Lys(Dabcyl)Ala-Lys(1-DeoxyFru)Ala-Phe-NH_2_ (**1**) to demonstrate the capturing properties of the novel PhB-Lys(PhB)-ChemMatrix^®^ Rink resin. Therefore, we built a calibration curve to discriminate, in mixtures, deoxyfructosylated peptides in presence of differently glycosylated peptides. In particular, peptide **1** contains the deoxyfructopyranosyl moiety linked to Lys3 side chain (already known to form esters with phenylboronic acid) [12], but also the 4-((4-(dimethylamino)phenyl)azo)benzoyl moiety (Dabcyl) linked to Lys1 side chain, exploiting its broad and intense visible absorption, to determine the minimal detectable concentration of the deoxyfructosylated model peptide **1** (Figures S6–S10). The synthetic building block Fmoc-Lys(Dabcyl)-OH (**I**) was used to prepare the calibration curves (Figures S11 and S12) and determine the concentration of the model peptide **1** before and after the capturing reaction. The maximum absorption of Fmoc-Lys(Dabcyl)-OH (**I**) in the capturing solution was ca. 445 nm, but in the cleavage solution the absorption was shifted to 455 nm (Figure S13).

To determine the maximum capacity of the PhB-Lys(PhB)-ChemMatrix^®^ Rink resin (substitution grade 0.28 mmol/g) in the capturing reaction conditions, we performed an experiment in saturating conditions of model deoxyfructosylated peptide **1** (Scheme S1). PhB-Lys(PhB)-CMRR (5 mg) was able to capture the model peptide **1** (0.0014 mmol), considering that only one active phenylboronic group attached to the lysine moiety could bind *cis* diol groups.

According to the obtained data, 5 mg of resin is capable to capture 1.11 mg of pure peptide. Therefore, the capturing efficiency of the proposed procedure is 81%. We proved that both the new resin and the regenerated resin batches have the same ability to capture the deoxyfructosylated peptide model **1** from the solution (Table 2).

Moreover, we determined the minimum time to dry the functionalised resin after synthesis obtaining a constant mass (Figure S14), showing that resin storage under atmospheric pressure causes insignificant changes in its weight (Figure S15).

### 3.2. The PhB-Lys(PhB)-ChemMatrix^®^ Rink Resin Is Specific for Deoxyfructosylated Peptides and Not for Differently Glycosylated Peptides

In order to assess the capturing efficiency of the PhB-Lys(PhB)-ChemMatrix^®^ Rink resin, a series of β-turn peptide structures of a model peptide termed CSF114 [28,29] were synthetically modified on the tip of the β-turn, with the amino acids Ser, Asn, and Lys at position 7 (u) bearing, on the side-chains by an *O*-glycosidic bond, amide, or amine bond, different glycosyl moieties (Table 3). The CSF114 peptide sequence (i.e., TPRVERu^7^GHSVFLAPYGWMVK) was selected for its optimised and previously demonstrated properties to expose at the best, at position 7, post-translational modifications [30]. The deoxyfructosylated peptide [(1-DeoxyFru)Lys7]CSF114 (**2**) was obtained, introducing during the solid-phase conditions of the synthesis, at position 7, the building block Fmoc-*L*-Lys(Boc)(2,3:4,5-di-*O*-isopropylidene-1-deoxyfructopyranosyl)-OH (**II**) [31]. The same approach was followed to synthesise [Asn^7^(Man)]CSF114 (**3**), [Asn^7^(Gal)]CSF114 (**4**), [Asn^7^(GlcNAc)]CSF114 (**5**), and [Asn^7^(Glc)]CSF114 (**6**) using the building blocks Fmoc-*L*-Asn[β-d-Man(OAc)_4_]-OH (**III**), Fmoc-*L*-Asn[β-D-Gal(OAc)_4_]-OH (**IV**), Fmoc-*L*-Asn[β-d-GlcNAc(OAc)_3_]-OH (**V**), and Fmoc-*L*-Asn[β-D-Glc(OAc)_4_]-OH (**VI**), respectively [32]. As control peptides, we synthesised the phosphorylated analogue [Ser^7^(PO_3_H_2_)]CSF114 (**7**) [33] (bearing the *O*-phosphoryl moiety on Ser7) and the corresponding non-glycosylated peptide CSF114 (**8**). We selected *O*-phosphorylation as control modification, because in previous studies we evaluated that dysregulation in protein expression can be involved in diabetes immune dysfunction [33].

According to the literature data, the furanose forms of the sugars display the best affinity for phenylboronic acid [35]. In particular, in solution, fructose is 25% in the β-d-fructofuranose form (with potentially reactive hydroxyl functions at positions 2, 3, and 6), galactose is 2.5% as α-d-galactofuranose (with potentially reactive hydroxyl functions at positions 2, 3 and 5, 6), while glucose is only 0.14% as α-d-glucofuranose (with potentially reactive hydroxyl functions at positions 1, 2 and 3, 5, 6). Moreover Kowalczyk et al. showed higher affinity and significant selectivity for the *N*-acetylneuraminic moiety over methyl-α-d-galactopyranoside, methyl-α/β-l-fucopyranoside, and *N*-acetyl-d-glucosamine [36]. In fact, the highest capturing affinity by PhB-Lys(PhB)-ChemMatrix^®^ Rink resin was observed with [(1-DeoxyFru)Lys^7^]CSF114 (**2**) (85% yield). The yield of the capturing reaction was estimated by the calibration curves reported in the SI (Figures S16–S18). [Asn^7^(Man)]CSF114 (**3**) was captured with a very low yield (5%) (Figure S19). On the other hand, the glycosylated peptides **4**–**6**, the *O*-phosphorylated peptide **7**, and the unglycosylated peptide CSF114 (**8**) did not react with CMRR (Figure S20). These results are the first insight that the deoxyfructopyranosyl moiety on lysine residues is crucial in the capturing reaction with the novel PhB-Lys(PhB)-ChemMatrix^®^ Rink resin (Figure S21).

### 3.3. The PhB-Lys(PhB)-ChemMatrix^®^ Rink Resin Is Specific for Deoxyfructosylated Peptides and Not for Unrelated Non-Glycosylated Peptides

We tested the binding efficiency of the PhB-Lys(PhB)-ChemMatrix^®^ Rink resin for deoxyfructosylated peptides as early glycation biomarkers of diabetes, in a peptide mixture mimetic of a digestion procedure of biological fluids. Therefore, we prepared three ad hoc aqueous solutions containing equimolar concentrations of all the synthetic peptides reported in Table 4 (1.4 μmol/mL), including the deoxyfructosylated peptide **2**: (i) at the same concentration (1.4 μmol/mL); (ii) 5% *w*/*w* (10.5 nmol/mL); (iii) 2% *w*/*w* (4.2 nmol/mL), to estimate the limit of detection of the deoxyfructosylated model peptide **2** by LC-MS. The ten synthetic peptides **7** and **9**–**17** (Table 4 and Table S2) were dissolved in an ammonium bicarbonate buffer solution at pH 8 and then subjected (as model matrix) to the capturing reaction conditions described above. These peptides were selected for the following characteristics: amino acid sequence, hydrophobicity, different length (from 4 to 35 amino acids) and post-translational modification (DeoxyFru, Ac, PO_3_H_2_, and Pam). The hexapeptide LSETTI (**11**) was selected as a previously developed peptide mimicking the HNK1 tri-saccharide epitope [37].

It is interesting to notice that Frolov et al. [19] previously reported that the efficiency of the *m*-aminophenylboronic acid (*m*-APBA) immobilised on agarose resin to capture Amadori peptides, after digestion of plasma protein from type 2 diabetes, was affected by unspecific recognition of peptide sequences containing several, often neighbouring, hydroxyamino acid residues. Therefore, in order to demonstrate the ideal and optimised capacity of the newly-functionalised PhB-Lys(PhB)-ChemMatrix^®^ Rink resin (herein proposed), only for deoxyfructosylated peptides, we selected the series of synthetic peptides containing those amino acid residues (Table 4). In addition, the tetrapeptide EKEK (**13**) was designed as a control sequence, because it contains only lysine residues (but no Tyr and Thr) that in diabetes are putative glycation motifs.

Our experiments demonstrated that the only peptide captured from the reaction mixture was the peptide [(1-DeoxyFru)Lys^7^]CSF114 (**2**). None of the non-deoxyfructosylated peptides were captured by the novel CMRR resin, demonstrating the high selectivity of our procedure for deoxyfructosylated peptides (Figures S22–S25). The lowest detectable concentration with proper signal-to-noise ratio is 5% (Figures S26 and S27).

### 3.4. The PhB-Lys(PhB)-ChemMatrix^®^ Rink Resin Is Also Specific for Deoxyfructosylated Peptides in Complex Peptide Mixtures (i.e., Human Serum Albumin and Bovine Serum Albumin Hydrolysates)

**Figure 1:**
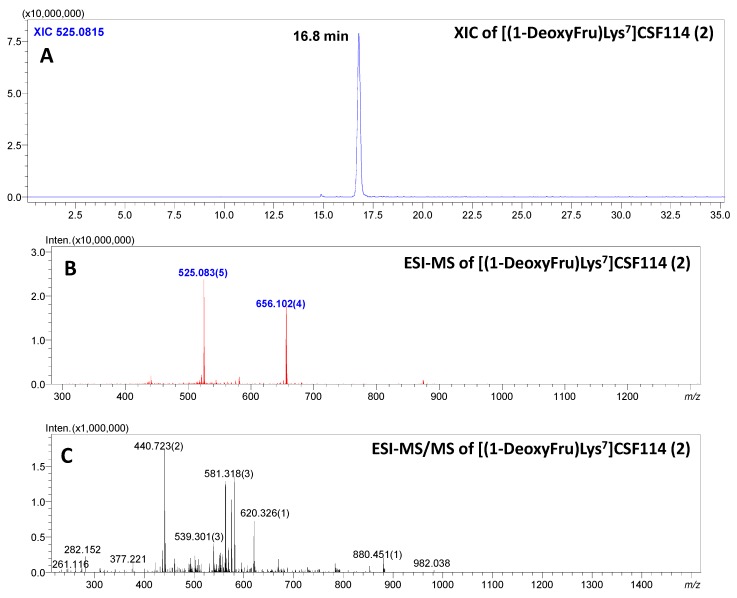
Analytical characterisation after performing the capturing procedure of the hydrolysate of Human Serum Albumin (HSA) with 2% *w/w* [(1-DeoxyFru)Lys7]CSF114 (**2**). Panel (**A**): Total Ion Chromatogram. Panel (**B**): ESI-MS. Panel (**C**): ESI-MS/MS.

The affinity of the PhB-Lys(PhB)-ChemMatrix^®^ Rink resin for the deoxyfructosylated peptides **2** and **18** was tested in complex hydrolysate mixtures of human and bovine serum albumin (HSA and BSA, respectively). Therefore, the above described procedure was applied to a mixture of the commercially available hydrolysate of HSA (97–99%, Sigma-Aldrich) in the presence of the deoxyfructosylated peptide **2** that was added: (i) at the same concentration (1.4 μmol/mL); (ii) 5% *w*/*w* (10.5 nmol/mL); or (iii) 2% *w*/*w* (4.2 nmol/mL) to estimate the limit of detection of the deoxyfructosylated peptide model **2** by LC-UV-MS (Figures S28 and S29). Interestingly, the most intensive signal detected corresponds to the deoxyfructosylated peptide model **2**. Detailed LC-MS/MS of the HSA hydrolysate containing peptide **2** at 5% *w*/*w* concentration, followed by bioinformatic analysis using the PEAKS software (see Materials and Methods), showed that only a negligible amount of two unrelated peptide sequences from HSA hydrolysate (ALVLIAFAQYLQQCPFEDHVK and DVFLGMFLYEYAR) were captured by CMRR (Figure S30). Interestingly, one of the sequences (including two tyrosine residues) was also reported by Frolov et al. [19] Moreover, the deoxyfructosylated peptide **2** at 2% *w*/*w* concentration was detectable only by LC-MS, but not by UV (Figure 1). At this concentration, no peptide sequence derived from the HSA hydrolysate could be detected (PEAKS software).

Unspecific interactions between the new functionalised resin and peptides derived from HSA hydrolysate were carefully investigated, applying the capturing procedure described above without addition of any deoxyfructosylated peptide. Detailed LC-MS analyses are presented in Figure S31. In particular, PEAKS bioinformatic analysis revealed that protein sequence coverage was only 16% and the seven low-intensity non-modified peptides identified (Figure S32) could be detected only if the deoxyfructosylated peptide **2** (besides having the highest affinity for the CMRR) is not present in the mixture and therefore, it cannot compete in binding.

**Figure 2:**
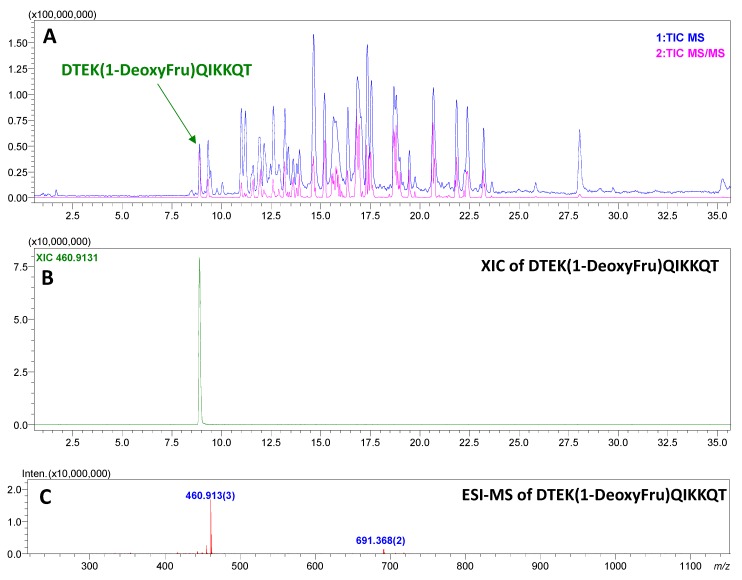
Analytical characterisation before performing the capturing procedure of the hydrolysate of Bovine Serum Albumin (500 pmol, BSA, BioLabs) with the deoxyfructosylated peptide DTEK(1-DeoxyFru)QIKKQT (**18**) (300 pmol). Panel (**A**): Total Ion Chromatogram. Panel (**B**): Extracted Ion Chromatogram. Panel (**C**): ESI-MS.

**Figure 3:**
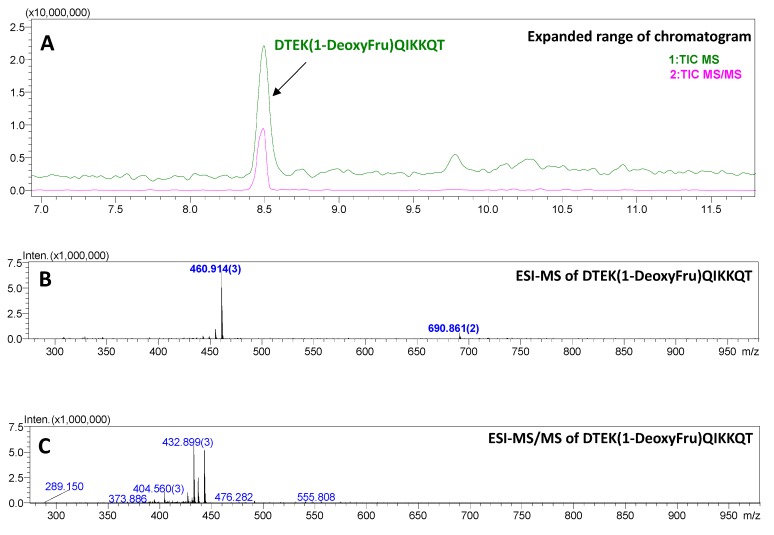
Analytical characterisation after performing the capturing procedure of the hydrolysate of Bovine Serum Albumin (500 pmol, BSA, BioLabs) with the deoxyfructosylated peptide DTEK(1-DeoxyFru)QIKKQT (**18**) (300 pmol). Panel (**A**): Total Ion Chromatogram, expanded range. Panel (**B**): ESI-MS. Panel (**C**): ESI-MS/MS.

An additional experiment was performed using the complex mixture of the commercially available BSA hydrolysate (500 pmol, BioLabs) in the presence of the analytically pure deoxyfructosylated BSA fragment DTEK(1-DeoxyFru)QIKKQT (**18**) (300 pmol). Before treating the complex mixture in the capturing reaction conditions, a detailed LC-MS/MS analysis was performed (Figure 2). Sequence coverage was 61% (Figure S33) and identity of the peptide DTEK(1-DeoxyFru)QIKKQT (**18**) was confirmed by XIC (Extracted Ion Chromatography), ESI-MS, and ESI-MS/MS (Figure 2). The concentration of the deoxyfructosylated peptide **18** was chosen unquestionably lower than the other peptides to display a lower mass intensity. Nevertheless, LC-MS/MS analysis revealed that the most intense signal (after reaction with the functionalised resin) corresponded to the deoxyfructosylated peptide **18** (Figure 3 and Figure S34). Moreover, bioinformatic analysis showed in BSA hydrolysate nine low intensity, but negligible peptide sequences, compared to peptide **18** (Figures S34 and S35). In conclusion, we demonstrated that decrease in deoxyfructosylated peptide concentration in the hydrolysate mixture might cause unspecific but insignificant interactions. Therefore, these unspecific but negligible interactions do not hamper the quality of the analysis, because the deoxyfructosyl-lysine containing the peptide **18** continues maintaining the highest affinity for the novel PhB-Lys(PhB)-ChemMatrix^®^ Rink resin presented herein and specifically-designed to trap deoxyfructosylated peptides to be efficiently detected by an enhanced mass spectrometry technique.

## 4. Conclusions

Deoxyfructosylation of peptides in human proteins is the early event of the complex glycation pathway typical of diabetes. Up to now, specific, selective, and high affinity early diagnostics of deoxyfructosylated peptides is always an unmet need for clinicians, to prevent further and, in most cases, fatal complications. In the present paper, we report the preparation, chemical characterisation, and capturing properties specific for deoxyfructosylated peptides, of a novel ChemMatrix^®^ Rink resin functionalised with two phenylboronate moieties (PhB) linked to the two amino functions of a lysine residue. The new PhB-Lys(PhB)-ChemMatrix^®^ Rink resin allows exploitation of the previously demonstrated ability of *cis* diols (present in the deoxyfructosyllysine moiety) to form phenylboronic esters. We demonstrated, by accurate ESI-MS analysis, that the novel PhB-Lys(PhB)-ChemMatrix^®^ Rink resin is stable to capturing reaction conditions. In fact, no signal corresponding to the possibly cleaved linker PhB-Lys(PhB)-NH_2_ was detected. Moreover, the resin loading is constant. Resin-capturing and -cleavage procedures were optimised to ensure that only the peptides containing deoxyfructosyllysine moieties can be efficiently and specifically detected by HR-MS and MS/MS experiments. The selectivity and affinity of the PhB-Lys(PhB)-ChemMatrix^®^ Rink resin was demonstrated in the case of three different peptides used in complex mixtures. First, we used, as a model, the simple synthetic pentapeptide H-Lys(Dabcyl)Ala-Lys(1-DeoxyFru)Ala-Phe-NH_2_ (**1**) containing Lys(Dabcyl), which, with its characteristic broad and intense visible absorption, allowed for the determination of the minimal detectable concentration. Using peptide **1**, the capturing efficiency of PhB-Lys(PhB)-ChemMatrix^®^ Rink resin was approximately 80%. It is worth highlighting that CMRR allows both the performance of orthogonal multidirectional synthesis, as well as accurate control by the specific PhB-Lys(PhB)-linker on the resin. The product can be removed from the resin at any time and can be characterised by analytical methods, which is not possible in the case of the agarose resin.

The significant binding of deoxyfructosylated peptides by functionalised CMRR, in comparison to other glycosylated peptides, may be explained considering the equilibrium of the different cyclic anomeric forms in solution, as reported in the literature (i.e., β-pyranose: ca. 75%; β-furanose: ca. 13%; and α-furanose: ca. 12%) [38,39]. Boronic acid is known to bind glucose preferentially in the α-furanose form and not in the more abundant α-pyranose form. β-D-Fructofuranose binds favourably to boronic acid because of the high presence of syn-periplanar hydroxyl groups [40]. This can explain the different binding constants to other sugars, such as galactose, mannose, or glucose. The binding of differently glycosylated and deoxyfructosylated peptides are strongly in agreement with data reported with free sugars, because only the deoxyfructosylated peptides bear the sugar in the furanose form. In the case of glycosylated peptides (e.g., galactosylated or glucosylated), only pyranose forms are present.

Moreover, we demonstrated that the regenerated resin has the same ability to capture the deoxyfructosylated peptide model **1** from the solution. Afterwards we investigated the high-selective affinity to deoxyfructosylated peptides **2** and **18** in an ad hoc mixture, containing also unique synthetic non-modified peptides, and in the hydrolysate of human and bovine serum albumin as an example of complex peptide mixture. We demonstrated that only peptide [(1-DeoxyFru)Lys^7^]CSF114 (**2**) was captured from the reaction mixture of synthetic non-modified peptides by the novel CMRR resin, showing unambiguously that the deoxyfructopyranosyl moieties on lysine residues are crucial in the capturing reaction. Testing the affinity of the PhB-Lys(PhB)-ChemMatrix^®^ Rink resin for different concentrations of deoxyfructosylated peptides **2** and **18** in complex hydrolysate mixtures of HSA and BSA, LC-MS/MS analysis showed that the most intense signal (after reaction with the functionalised resin), corresponded to deoxyfructosylated peptide **18** (or **2**). PEAKS bioinformatics analysis revealed that decreasing the concentration of deoxyfructosylated peptides in the hydrolysate mixture might cause unspecific, but insignificant interactions, because intensity of the identified peptides originating from hydrolysate was low compared with the deoxyfructosyl-lysine containing peptides (**18** or **2**). Therefore, the novel specifically-designed PhB-Lys(PhB)-ChemMatrix^®^ Rink resin, displaying highest affinity to deoxyfructosylated peptides, is a candidate to quantitatively separate early glycation peptides from complex mixtures to investigate their role in diabetes complications.

## Figures and Tables

**Table 1:** Loading of PhB-Lys(PhB)-ChemMatrix^®^ Rink resin (M = 0.28 mmol/g, SD = 0.007).

PhB-Lys(PhB)-CMRR (mg)	Sample Dilution	Abs (OD)	n [mmol]	PhB-Lys(PhB)-CMRR Loading (mmol/g)
2.91	50	0.4784	8.18 × 10^−4^	0.281
3.50	50	0.5758	9.73 × 10^−^^4^	0.278
5.40	100	0.4552	1.56 × 10^−3^	0.289
6.07	50	0.9892	1.63 × 10^−3^	0.269
5.00	50	0.8333	1.38 × 10^−3^	0.277

**Table 2:** Comparison of capturing efficiency of new and reused (once or twice) batches of the PhB-Lys(PhB)-ChemMatrix^®^ Rink resin.

Results	PhB-Lys(PhB)-CMRR		After First Use of PhB-Lys(PhB)-CMRR		After Second Use of PhB-Lys(PhB)-CMRR	
	Unreacted	Released from Resin	Unreacted	Released from Resin	Unreacted	Released from Resin
Absorbance (OD)	0.1436	0.6670	0.1498	0.5602	0.3069	0.4872
n (nmol)	0.200	0.531	0.125	0.446	0.260	0.388
mass (mg)	0.117	0.519	0.122	0.435	0.252	0.375
Capturing yield (%)	81		78		60	
Reused batch (%)	-		96		73	

**Table 3:** Model peptides **2**–**7** based on the β-turn structure CSF114 (**8**) bearing, at position 7, different post-translational modifications.

Glycopeptide	Building Block
[(1-DeoxyFru)Lys^7^]CSF114 (**2**)	Fmoc-*L*-Lys(Boc)(2,3:4,5-di-*O*-isopropylidene-1-deoxyfructopyranosyl)-OH (**II**) ^a^
[Asn^7^(Man)]CSF114 (**3**)	Fmoc-*L*-Asn[β-d-Man(OAc)_4_]-OH (**III**) ^b^
[Asn^7^(Gal)]CSF114 (**4**)	Fmoc-*L*-Asn[β-d-Gal(OAc)_4_]-OH (**IV**) ^b^
[Asn^7^(GlcNAc)]CSF114 (**5**)	Fmoc-*L*-Asn[β-d-GlcNAc(OAc)_3_]-OH (**V**) ^c^
[Asn^7^(Glc)]CSF114 (**6**)	Fmoc-*L*-Asn[β-d-Glc(OAc)_4_]-OH (**VI**) ^b^
[Ser^7^(PO_3_H_2_)]CSF114 (**7**)	Fmoc-*L*-Ser(PO_3_Bzl,H)-OH (**VII**) ^c^
CSF114 (**8**)	Fmoc-*L*-Asn(Trt)-OH (**VIII**) ^c^

^a^ [31]; ^b^ [34]; ^c^ commercially available building blocks (Merck).

**Table 4:** Synthetic peptides used as a matrix for capturing [(1-DeoxyFru)Lys^7^]CSF114.

N	Peptide
**2**	[(1-DeoxyFru)K^7^]TPRVERGHSVFLAPYGWMVK
**9**	Ac-GKNAT
**10**	Ac-MSKVVNPTQK-NH_2_
**11**	LSETTI
**12**	DQDAEQAPEYRGRTELLKET
**13**	EKEK
**14**	REKLVVRRGQPFWLTLHFEGR
**7**	[(PO_3_H_2_)S^7^]TPRVERGHSVFLAPYGWMVK
**15**	GQFRVIGPGYPIRALVGDEAELPCRISPGKNATG
**16**	ENPVVHFFKNIVTPRTP
**17**	Pam-ENPVVHFFKNIVTPRT

Ser, Tyr, and Thr highlighted in red; Lys highlighted in blue; post-translational modifications (DeoxyFru, Ac, PO_3_H_2_, and Pam) highlighted in green.

## Supplementary Materials

The following are available online: Details of the synthetic procedures, technical details for the analytical procedures, i.e., UV-VIS, ESI-MS, ESI-MS/MS, LC-MS, LC-UV-MS, and LC-MS/MS spectra and PEAKS bioinformatics data analyses.
