# Supplementary material for: An Optimised Di-Boronate-ChemMatrix Affinity Chromatography to Trap Deoxyfructosylated Peptides as Biomarkers of Glycation
Source: Molecules. 2020 Feb 10;25(3):755. doi: 10.3390/molecules25030755 (PMC7037614; doi:10.3390/molecules25030755)
Supplement: Supplementary file 1 [file molecules-25-00755-s001.pdf]

An Optimised Di-Boronate-ChemMatrix Affinity Chromatography to Trap Deoxyfructosylated Peptides as Biomarkers of Glycation

Monika Kijewska,[1] Francesca Nuti,[2] Magdalena Wierzbicka,[1] Mateusz Waliczek,[1] Patrycja Ledwoń,[1,3] Agnieszka Staśkiewicz,[1,2] Feliciana Real-Fernandez,[2,3] Giuseppina Sabatino,[2,4] Paolo Rovero,[3] Piotr Stefanowicz,[1] Zbigniew Szewczuk,[1] Anna Maria Papini.[2,5]

[1]Faculty of Chemistry University of Wrocław, ul. F. Joliot-Curie 14, 50-383 Wrocław, Poland.
[2]Laboratory of Peptide and Protein Chemistry and Biology, Department of Chemistry "Ugo Schiff", University of Florence, Via della Lastruccia 13, 50019, Sesto Fiorentino, Italy.
[3]Laboratory of Peptide and Protein Chemistry and Biology, Department of Neurosciences, Psychology, Drug Research and Child Health - Section of Pharmaceutical Sciences and Nutraceutics, University of Florence, Via Ugo Schiff 6, 50019 Sesto Fiorentino, Italy.
[4]CNR-IC Istituto di Cristallografia, Via Paolo Gaifami 18, 95126 Catania, Italy
[5]PeptLab@UCP and Laboratory of Chemical Biology EA4505, CY Cergy Paris University, 5 Mail Gay-Lussac, 95031 Cergy-Pontoise, France.

Corresponding authors: Monika Kijewska, *e-mail*: monika.kijewska@chem.uni.wroc.pl

Anna Maria Papini, *e-mail:* annamaria.papini@unifi.it

SYNTHETIC PROCEDURES

General Procedure

*Synthesis of Functionalized Resin PhB-Lys(PhB)-ChemMatrix® Rink Resin and of the Model Deoxyfructosylated Peptide (**1**):* The amino acid derivatives for peptide synthesis, including Fmoc-Lys(Dabcyl)-OH, Fmoc-Lys(Fmoc)-OH, and the coupling reagents TBTU (O-(Benzotriazol-1-yl)-*N,N,N',N'*-tetramethyluronium tetrafluoroborate), PyBop (benzotriazol-1-yl-oxytripyrrolidinophosphonium hexafluorophosphate) were purchased from NovaBiochem. The ChemMatrix® Rink Resin (CMRR) (0.40–0.60 mmol/g) was purchased from Aldrich. The solvents for peptide synthesis (analytical grade) were obtained from Riedel de Haën (DMF) and J. T. Baker (methanol). Other solvents used in this work were obtained from Aldrich. All reagents and solvents were used as purchased without further purification. Human Serum Albumin and Trypsin (TPCK – from bovine pancreas) were purchased from Sigma-Aldrich. Tryptic digested Bovine Serum Albumin (BSA) was purchased from BioLabs.

Synthesis of the Functionalized PhB-Lys(PhB)-ChemMatrix® Rink Resin

The ChemMatrix® Rink resin (loading 0.4-0.6 mmol/g) was swelled in DMF for 30 min (90 mg, 0.054 mmol). Fmoc-Lys(Fmoc)-OH (96 mg, 0.16 mmol) was added and linked by TBTU (52 mg, 0.16 mmol) as a coupling reagent in the presence of DIEA (60 µL, 0.32 mmol) in 2 h. The reaction was controlled by ninhydrin test. After Fmoc deprotection from *N*-terminus and ε-amino group of lysine by 25% piperidine in DMF, the resin was washed with DMF (7 × 1 min). The 4-carboxyphenylboronic acid (PhB-OH) (54 mg, 0.32 mmol) was incorporated using PyBOP (168 mg, 0.32 mmol) as a coupling reagent and DIEA (120 µL, 0.64 mmol) in 12 h. After the reaction was completed the resin was washed with: DMF (7 × 1 min), DCM (3 × 1 min), THF (3 × 1 min), MeOH (3 × 1 min), and Et_2O (3 × 1 min). of the functionalised resin was dried under *vacuum* for three days at room temperature. PhB-Lys(PhB)-NH_2 was cleaved from the resin using a mixture of TFA/H_2O/TIS 95:2.5:2.5 *(v:v:v)* in 2 h. The solution was evaporated under gentle stream of nitrogen and then lyophilized.

*Solid-Phase Synthesis of the Model Deoxyfructosylated Peptide H-K(Dabcyl)AK(DeoxyFru)AF-NH_2 (**1**)*

The model peptide (**1**) was synthesized according to the standard Fmoc/tBu procedure on a ChemMatrix® Rink resin (loading 0.4-0.6 mmol/g) (CMRR), using the commercially available protected amino acids Fmoc-Phe-OH, Fmoc-Ala-OH, Fmoc-Lys(Boc)-OH, Fmoc-Lys(Dabcyl)-OH, and Fmoc-*L*-Lys(Boc)(2,3:4,5-di-O-isopropylidene-1-deoxyfructopyranosyl)-OH (**II**) that was synthesized according to the previously reported procedure [*Carganico S, Rovero P, Halperin JA, Papini AM, Chorev M Building blocks for the synthesis of post-translationally modified glycated peptides and proteins. J Pept Sci 2009, 15, 67-71*]. TBTU was used as a coupling reagent in the presence of DIEA. After the reaction was completed the resin was washed: DMF (7×1 min), DCM (3×1 min), THF (3×1 min), MeOH

(3×1 min) and Et_2O (3×1 min). Then the resin was dried under *vacuum* for three days at room temperature. The peptide was cleaved from the resin using a mixture of TFA/H_2O/TIS 95:2.5:2.5 (*v/v/v*) for 8 h at room temperature and precipitated with cold diethyl ether.

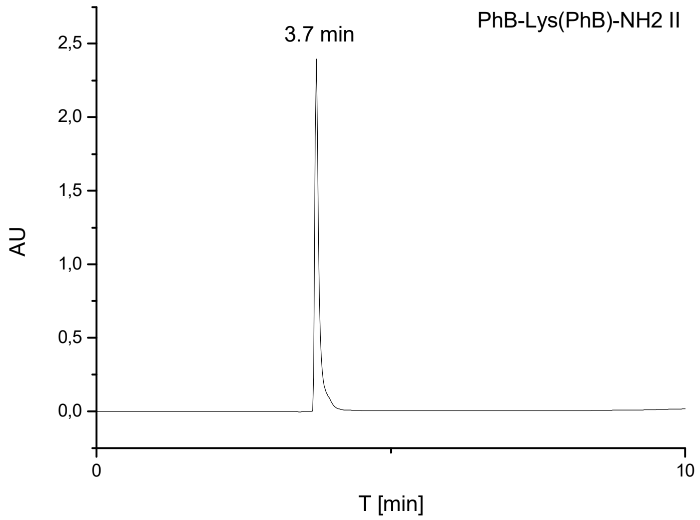

Fig. S1 HPLC of PhB-Lys(PhB)-NH_2

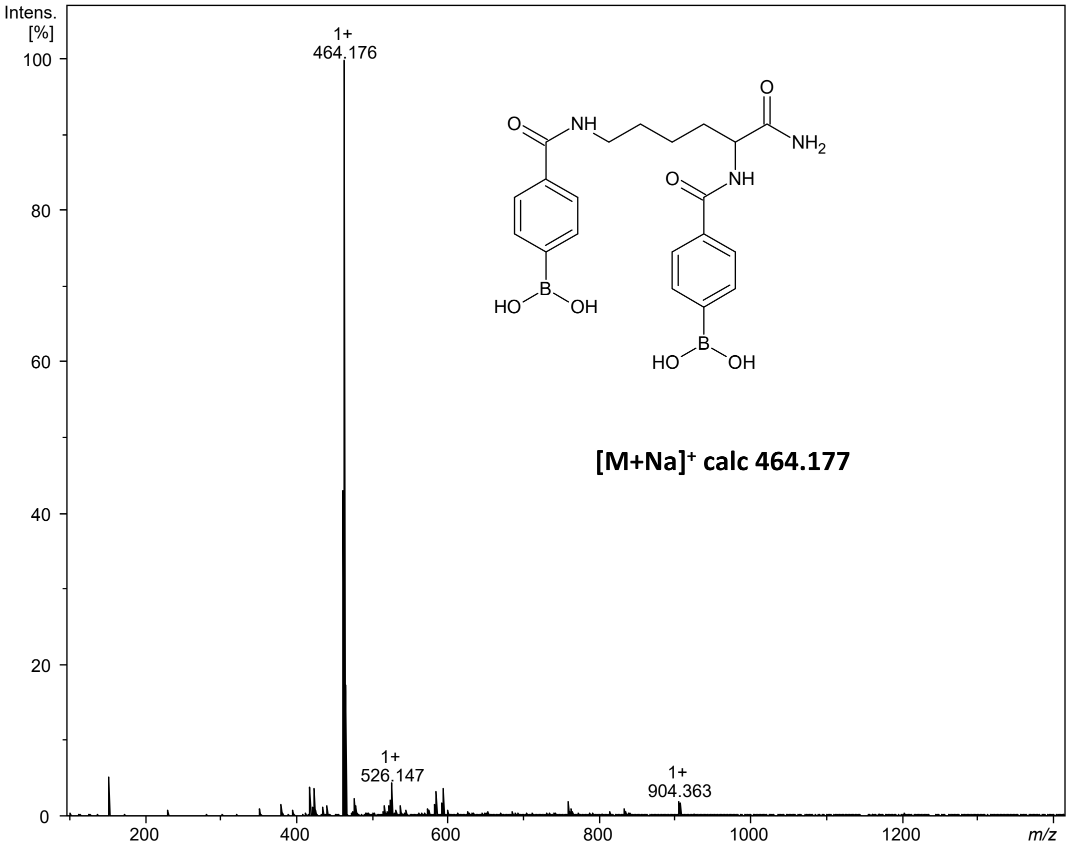

Fig. S2 ESI-MS spectrum of PhB-Lys(PhB)-NH_2 (MS value *m/z* =464.176) obtained after acidic hydrolysis of PhB-Lys(PhB)-CMRR (ESI-FT-MS, solvent: methanol with Sodium ion. Experimental details are reported in the text.

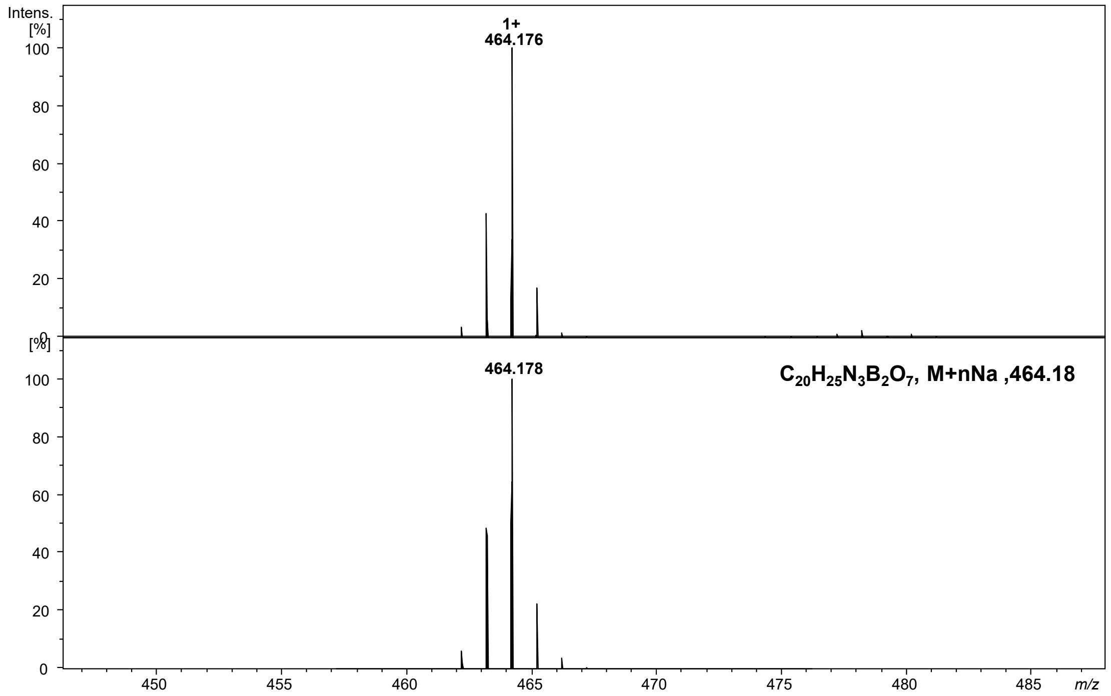

Fig. S3 ESI-MS spectrum of PhB-Lys(PhB)-NH_2 performed in NaCl 10 mM in methanol. Bottom panel: simulated isotopic pattern of the molecular formula.

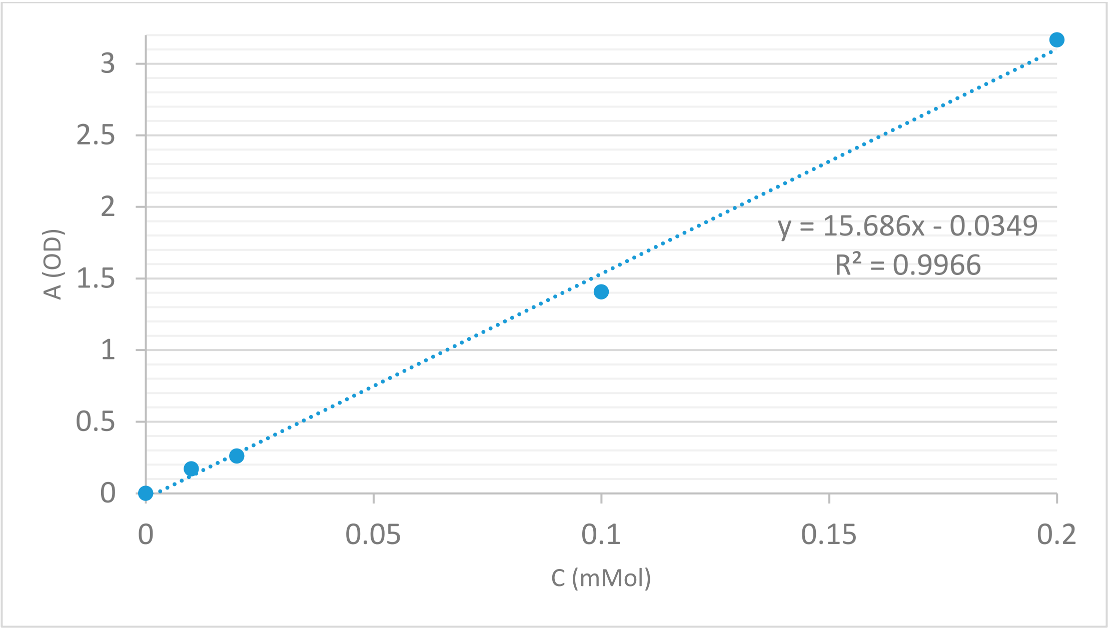

Fig. S4 Calibration curve of 4-carboxyphenylboronic acid (PhB-OH, Sigma-Aldrich) with reported the calibration equation.

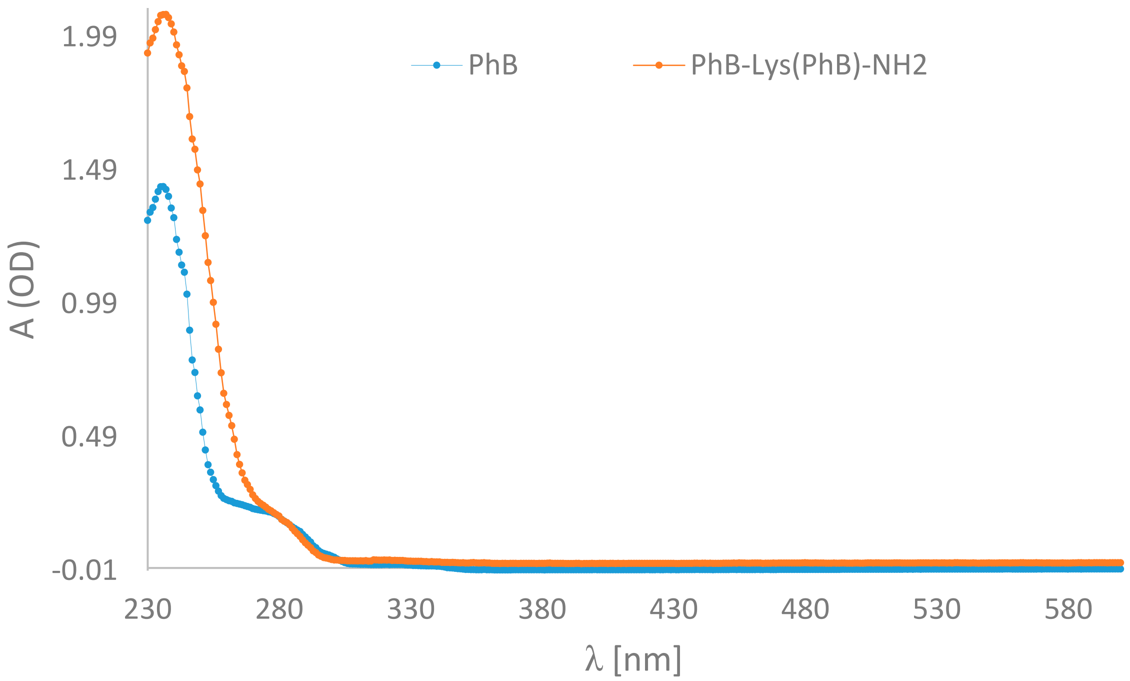

Fig. S5 UV-VIS spectra of 4-carboxyphenylboronic acid (PhB-OH, Sigma-Aldrich) and crude PhB-Lys(PhB)-NH_2 after cleavage from PhB-Lys(PhB)-CMRR measured by Tecan Infinite Plate Reader.

The peptide was purified by HPLC and analyzed by ES-MS and ESI-MS/MS. The ESI-MS spectrum of pure H-K(Dabcyl)AK(1-DeoxyFru)AF-NH_2 peptide (**1**) is presented in **Fig. S6**. The characteristic neutral losses are observed. The fragmentation spectrum (**Fig. S7**) confirmed the structure of the peptide (**1**). The purity of the peptide (**1**) is shown in **Fig. S8**. LC-MS with PDA analysis was performed for pure H-K(Dabcyl)AK(1-DeoxyFru)AF-NH_2 (**1**). In **Fig. S9** the signal corresponding to $[M+2H]^2+$ of peptide (**1**) is observed. The same signal is also presented in 3D projection (**Fig. S10**). Moreover, MRM analysis was performed using three transition pairs 488.5 > 252.02 (dabcyl moiety); 488.5 > 461.7 $(M-3H_2O+2H]^2+)$ and 488.5 > 446.65 $(M-3H_2O-HCHO+2H]^{2+})$ *m/z*. The retention time is 4.6 min.

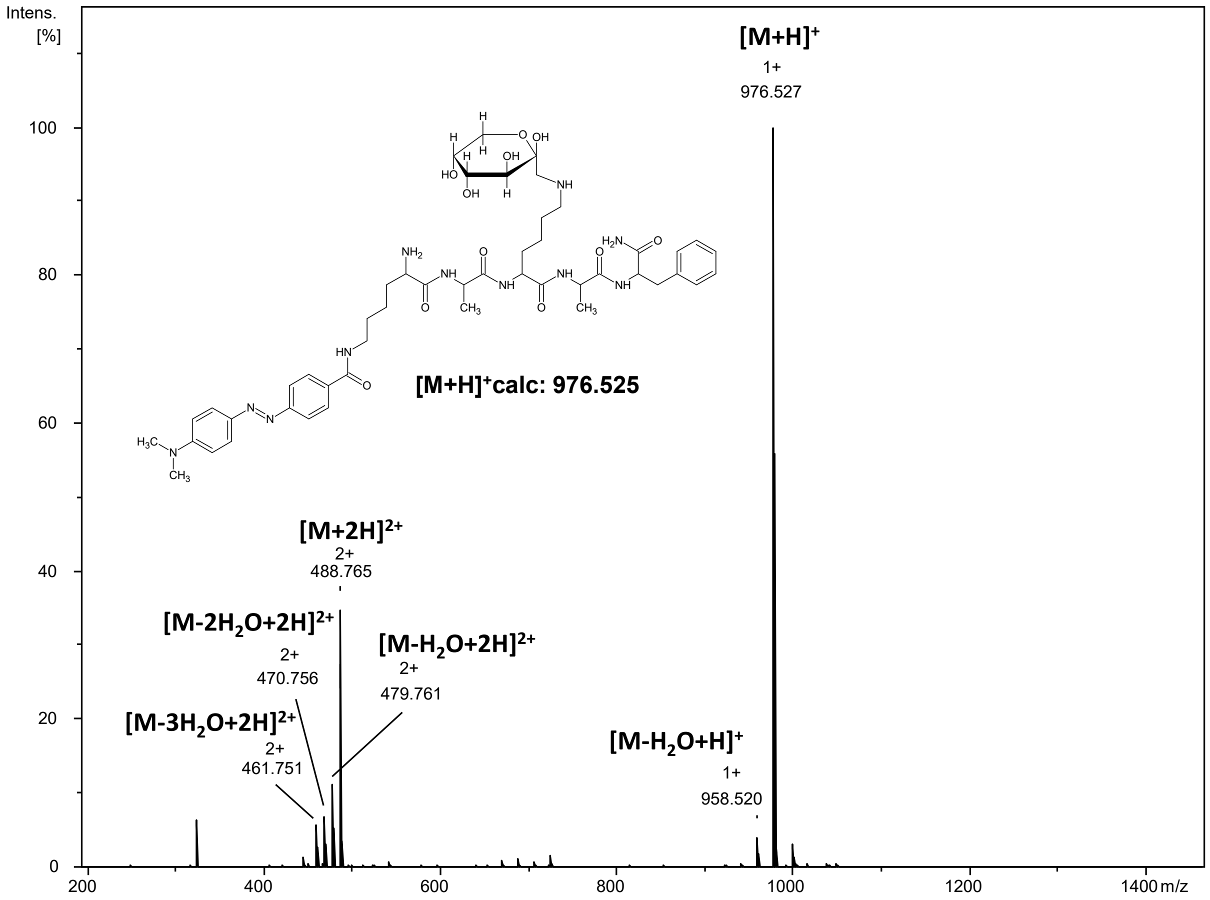

Fig. S6 ESI-MS of pure H-K(Dabcyl)AK(1-DeoxyFru)AF-NH_2 (**1**)

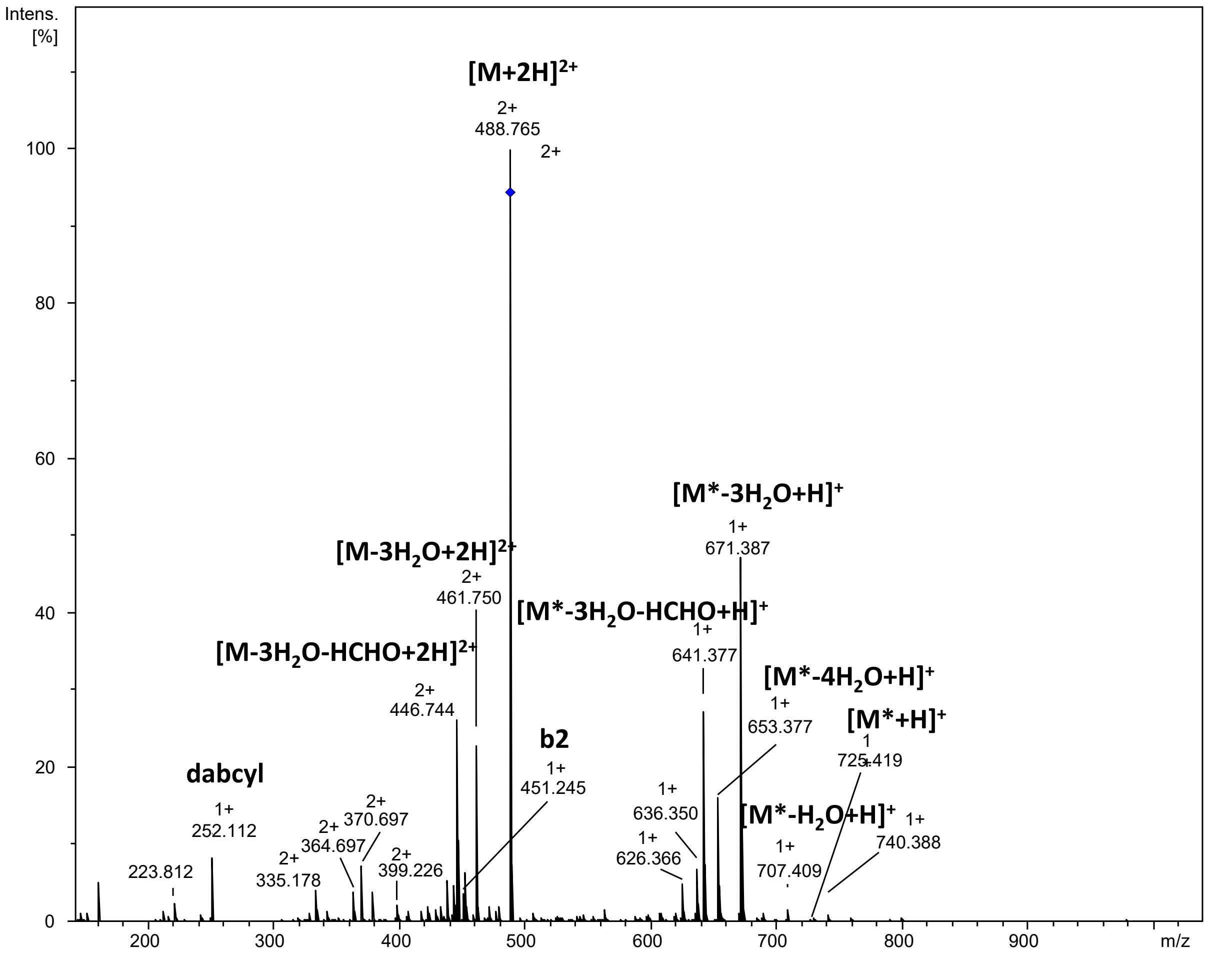

Fig. S7 ESI-MS/MS of pure H-K(Dabcyl)AK(1-DeoxyFru)AF-NH_2 (**1**) (parent ion 488.76; collision energy 25 eV) (M* = M without the Dabcyl moiety)

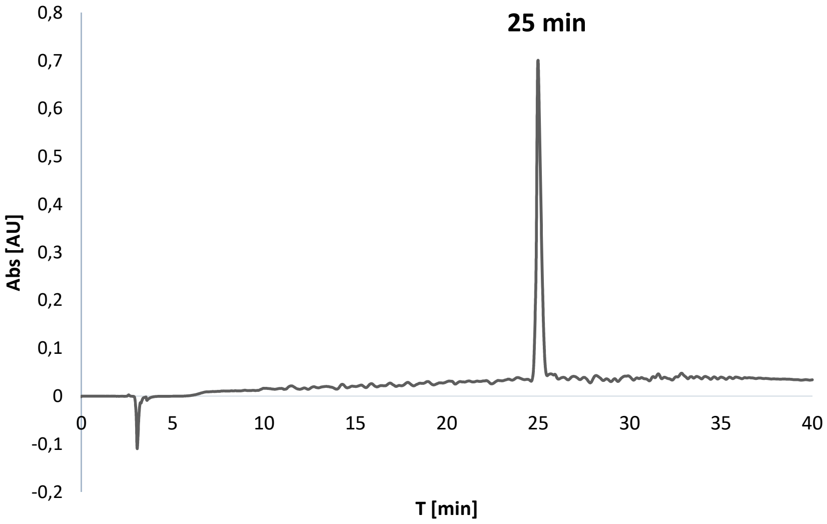

Fig. S8 Chromatogram of pure H-K(Dabcyl)AK(1-DeoxyFru)AF-NH_2 (**1**)

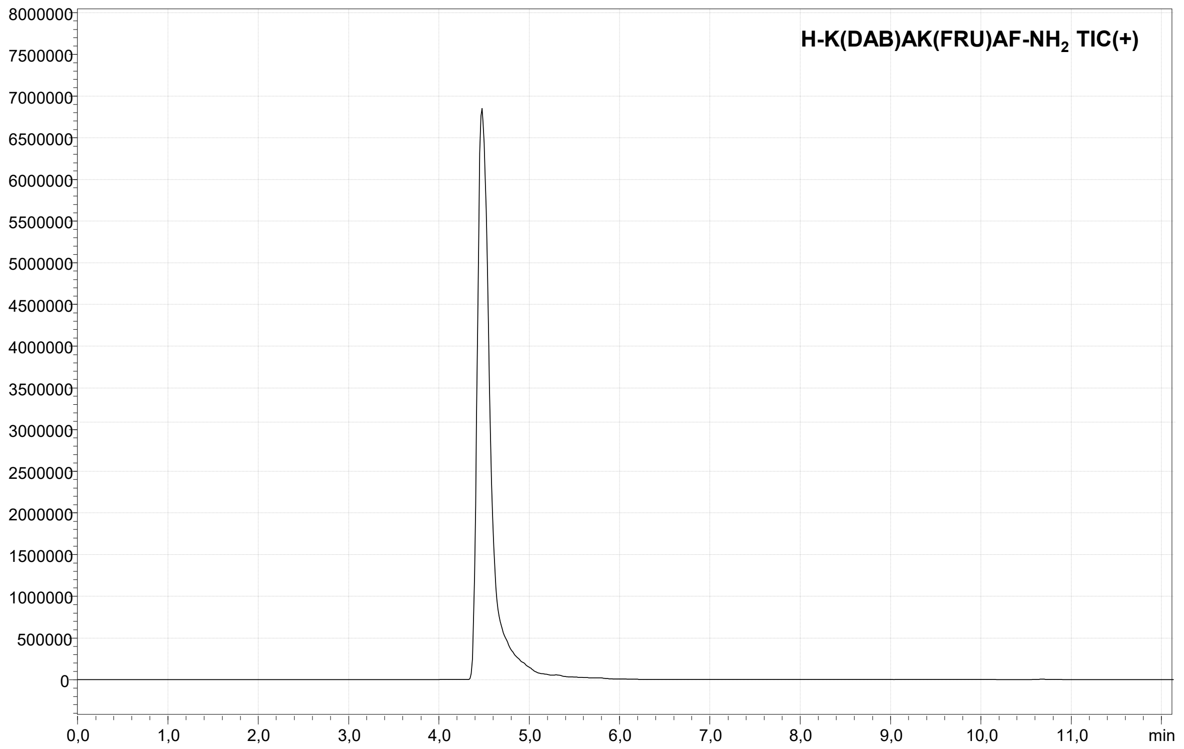

Fig. S9 LC-MS of H-K(Dabcyl)AK(1-DeoxyFru)AF-NH2 (**1**) (TIC: Total Ion Current)

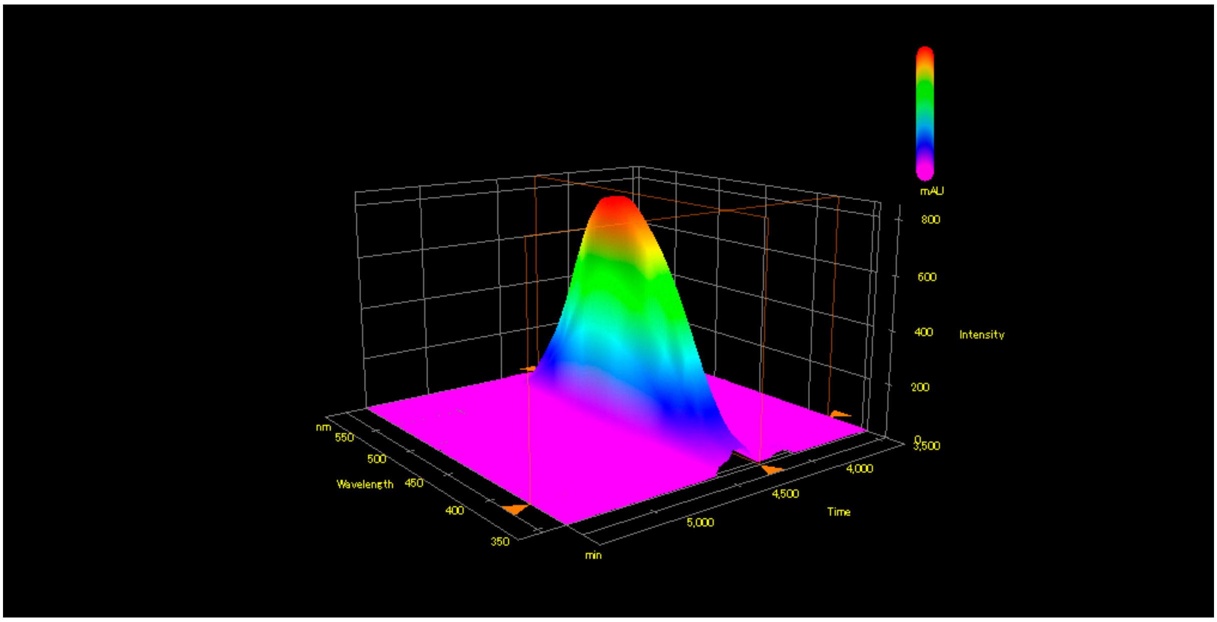

Fig. S10 3D analysis of the signal corresponding to H-K(Dabcyl)AK(1-DeoxyFru)AF-NH2 (**1**) (max Abs 455 nm).

The commercially available building-block Fmoc-Lys(Dabcyl)-OH (**I**) was used to prepare two different calibration curves: one for the capturing conditions, i.e., ammonium bicarbonate buffer solution at pH 8, and for the cleavage solution, i.e., H_2O/MeCN 1:1 (*v/v*) containing 0.1% HCOOH (**Fig. S11, Fig. S12**). The maximum absorption of Fmoc-Lys(Dabcyl)-OH (**I**) in the capturing solution was ca. 445 nm and in the cleavage solution was shifted to 455 nm (**Fig.**

S13). The concentration of the model peptide **1** used to optimise the capturing reaction was determined on the basis of the calibration curve in the buffer solution, whereas the concentration of the peptide separated from the mixture was established on the basis of the calibration curve prepared in the cleavage solution. The content of the pure peptide **1** after lyophilisation was evaluated 90% in weight.

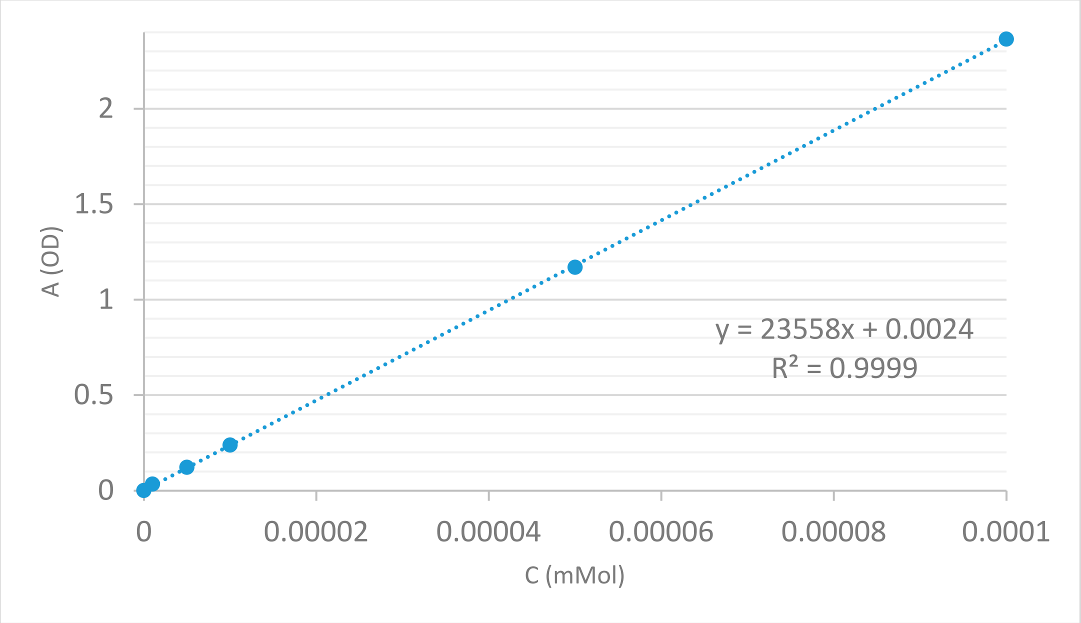

Fig. S11 Calibration curve of Fmoc-Lys(Dabcyl)-OH (**I**) in ammonium bicarbonate buffer with reported the calibration equation.

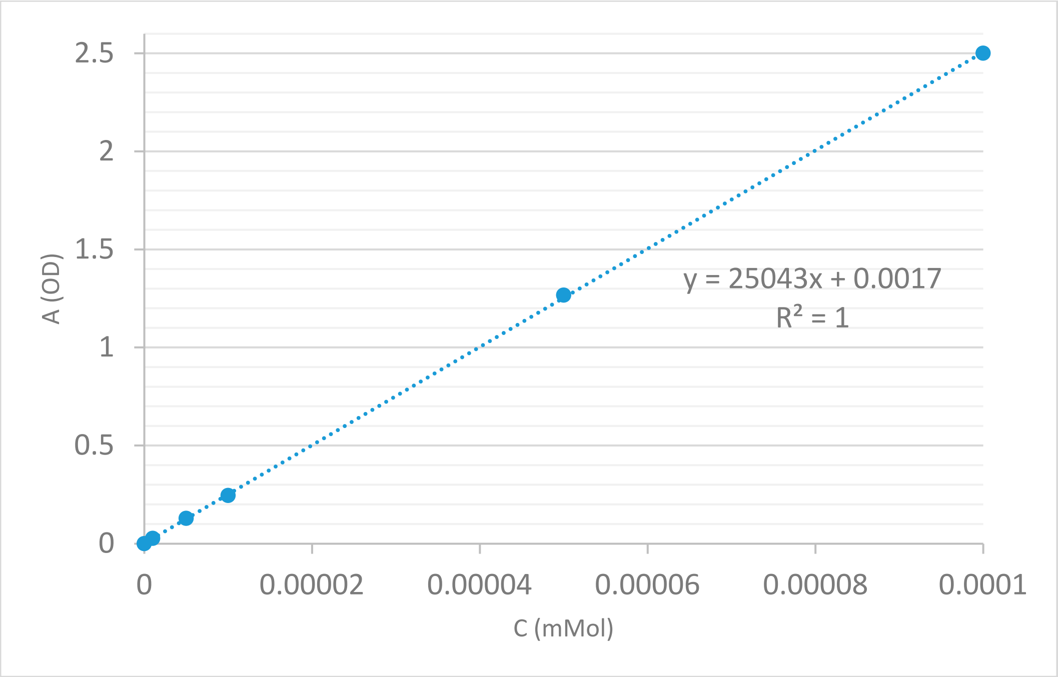

Fig. S12 Calibration curve for Fmoc-Lys(Dabcyl)-OH (**I**) in ammonium bicarbonate buffer. The calibration equation is also reported.

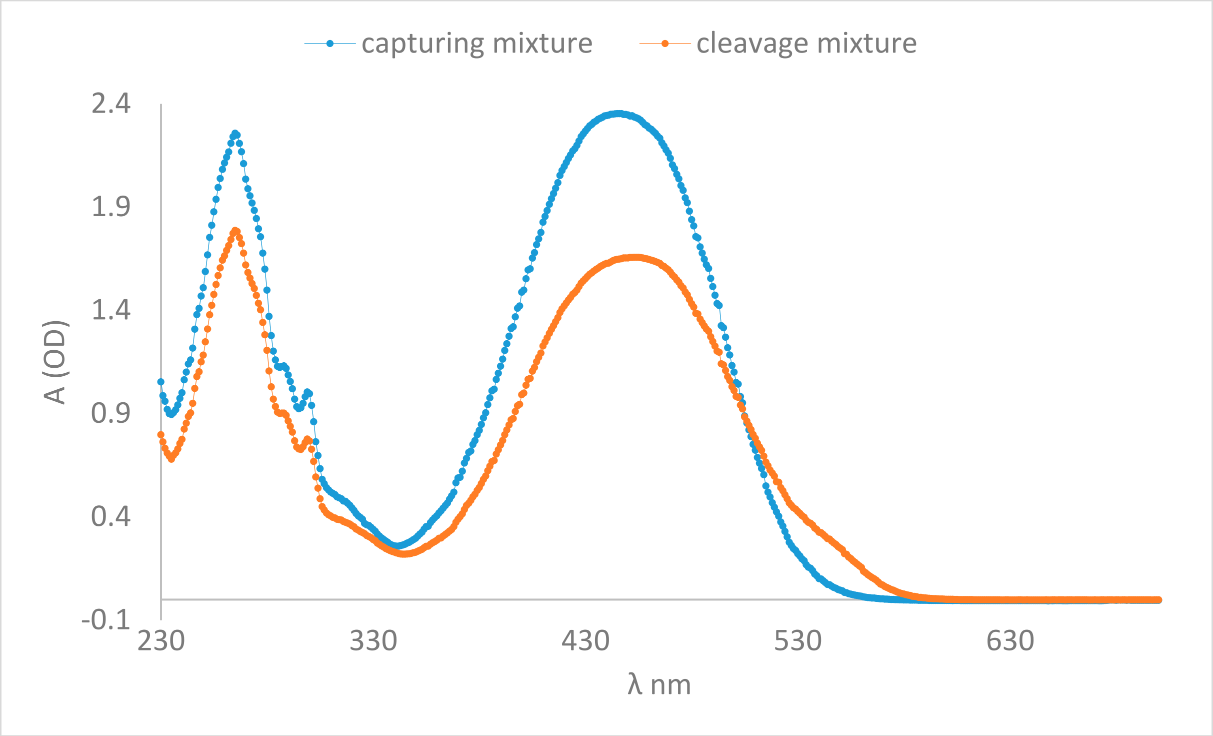

Fig. S13 UV-VIS spectra of Fmoc-Lys(Dabcyl)-OH (NovaBiochem) in: capturing mixture (blue line) and cleavage mixture (orange line), measured by Tecan Infinite Plate Reader

*MW-Assisted Solid Phase Synthesis of the β-turn glycopeptide structures (**2**-**7**)*

All Fmoc-protected amino acids, Fmoc-Wang resins, DIC (*N,N'*-Diisopropylcarbodiimide), and Oxyma were purchased from Iris Biotech GmbH (Marktredwitz, Germany). Fmoc-*L*-Asn[β-D-GlcNAc(OAc)$_3$]-OH (**V**) and Fmoc-*L*-Ser(PO$_3$Bzl,H)-OH (**VII**) were purchased from Bachem (Germany). Fmoc-*L*-Lys(Boc)(2,3:4,5-di-*O*-isopropylidene-1-deoxyfructopyranosyl)-OH (**II**) was prepared as previously described [*Carganico S, Rovero P, Halperin JA, Papini AM, Chorev M Building blocks for the synthesis of post-translationally modified glycated peptides and proteins. J Pept Sci 2009, 15, 67-71*]. Fmoc-L-Asn[β-D-Man(OAc)$_4$]-OH (**III**), Fmoc-*L*-Asn[β-D-Gal(OAc)$_4$]-OH (**IV**), Fmoc-*L*-Asn[β-D-Glc(OAc)$_4$]-OH (**VI**) were synthesized as previously described [*Paolini I, Nuti F, Pozo-Carrero MC, Barbetti F, Kolesinska B, Kaminski ZJ, Chelli M, Papini AM. A convenient microwave-assisted synthesis of N-glycosyl amino acids, Tetrahedron Letters 2007, 48(16), 2901-2904*].

The β-turn glycopeptide structures were synthesized by microwave-assisted solid-phase synthesis (MW-SPPS) following the Fmoc/tBu strategy, using the Liberty Blue™ automated microwave peptide synthetizer (CEM Corporation, Matthews, NC, USA) following the protocol previously described [*Rizzolo F, Testa C, Lambardi D, Chorev M, Chelli M, Rovero P, Papini AM. Conventional and microwave-assisted SPPS approach: a comparative synthesis of PTHrP(1-34)NH$_2$. J Pept Sci 2011, 17(10), 708-14*].

The resin used was a Fmoc-Lys(Boc)-Wang (loading 0.24 mmol/g). Couplings were performed using the adequately protected amino acids (2.5 eq), HATU as activator (2.5 eq), and DIPEA (3.5 eq) in 30 min at room temperature.

Each coupling step was monitored by the Kaiser test [*Kaiser E, Colescott RL, Bossinger CD, P.I. Cook PI. Color test for detection of free terminal amino groups in the solid-phase synthesis of peptides, Anal Biochem 1970, 34(2), 595-598*] or micro-cleavages performed with a microwave apparatus CEM Discover™ single-mode MW reactor (CEM Corporation, Matthews, NC, USA). Final cleavages were performed using a mixture of TFA/TIS/H_2O 95:2.5:2.5 (*v:v:v*) for 3 hours at room temperature.

Deprotection of the hydroxyl functions of the sugar moieties linked to the *O*-glycosylated peptides was performed with a 0.1 M NaOMe solution in MeOH until pH 11-12 added to a solution of the lyophilized peptides in dry MeOH (1mL/100mg of resin) under N_2 atmosphere. Reaction was monitored by UPLC-MS every 20 min to control until the deprotection step was completed. Once reaction was complete, the mixture was quenched by adding concentrated HCl until pH 7, the solvent was evaporated under *vacuum* and the residue lyophilized.

Purification of the synthetic peptides was performed by semipreparative RP-HPLC on a Waters instrument (Sepation Module 2695, detector diode array 2996) using a Phenomenex (Torrance, CA, USA) Jupiter column C18 (10 µm, 250×10 mm), at 4 mL/min with solvent system A (0.1% TFA in H_2O) and B (0.1% TFA in CH_3CN). Characterization of the peptides was performed by analytical UPLC using a Waters ACQUITY UPLC coupled to a single quadrupole ESI-MS (Waters 3100 Mass Detector) supplied with a BEH C18 (1.7 µm 2.1× 50 mm) column at 35 °C, at 0.6 mL/min with solvent system A (0.1% TFA in H_2O) and B (0.1% TFA in CH_3CN). The peptides were purified by semi-preparative RP-HPLC and characterized by RP-HPLC ESI-MS, obtaining a final purity ≥ 98%.

Table S1: Analytical data of the synthetic peptides **2-8**

**Peptide**	**ESI-MS (m/z) (Exact Mass calcd)[a] found[b]**	**HPLC (t_R, min)**
[(1-DeoxyFru)Lys^7]CSF114 (**2**)	(2620.4) 1311.2	3.99^c
[Asn^7(Man)]CSF114 (**3**)	(2606.3) 1304.7	2.38^c
[Asn^7(Gal)]CSF114 (**4**)	(2606.3) 1304.13	4.18^c
[Asn^7(GlcNAc)]CSF114 (**5**)	(2647.2) 1324.6	4.17^c
[Asn^7(Glc)]CSF114 (**6**)	(2606.3) 1303.8	4.12^c
[Ser^7(PO_3H_2)]CSF114 (**7**)	(2499.2) 1249.0	4.02^c
CSF114 (**8**)	(2445.6) 1223.33	4.23

^{a}ESI-MS: detected as ^{a}[M+H]$^+$; ^{b}[M+2H]$^{2+}$. Solvent system A: 0.1% TFA in H_2O, B: 0.1% TFA in CH_3CN. Analytical HPLC gradients at 0.6 mL min^{-1}: ^{c} 10-90% B in 5 min.

ANALYTICAL METHODS

HPLC analysis

HPLC analysis of PhB-Lys(PhB)-NH_2 and PhB-OH were carried out on Thermo Separation HPLC system with a UV detection (240 nm) and a Vydac Protein RP C18 column (4.6 × 250 mm, 5 µm), with a gradient elution of 0%–30% S2 in S1 (S1 = 0.1% aqueous TFA in H_2O; S2 = 80% acetonitrile + 0.1% TFA) for 30 min (flow rate: 1 mL/min at RT).

ESI-MS experiments

The ESI-MS experiments were performed using an Apex-Qe 7T instrument (Bruker) equipped with a dual ESI source. The acetonitrile/water/formic acid (50:50:0.1) mixture or methanol were used as solvents for recording the mass spectra. The potential between the spray needle and the orifice was set to 4.5 kV. In the MS/MS mode, the quadrupole was used to select the precursor ions, which were fragmented in the hexapole collision cell applying argon as a target gas. The obtained fragments were subsequently mass analysed by the ICR mass analyser. For CID MS/MS measurements, the voltage 20 V over the hexapole collision cell was applied.

HPLC-MS analysis

For the model deoxyfructosylated peptide H-K(Dabcyl)AK(1-DeoxyFru)AF-NH_2 (**1**): The HPLC-MS analysis was performed on Shimadzu LC MS-8050 equipped with a triple quadrupole mass spectrometer using MRM (Multiple Reaction Monitoring) mode and Q1Q3 scan. Separation was carried out on an RP-Zorbax (50×2.1 mm, 3.5 µm) column with a gradient elution of 0-30% B in A (A = 0.1% HCOOH in water; B = 0.1% HCOOH in MeCN) at room temperature over a period of 12 min (flow rate: 0.1 mL/min). High resolution mass spectra were measured using an Apex-Qe 7T instrument (Bruker) equipped with a dual ESI source.

For the deoxyfructosylated peptide [(1-DeoxyFru)Lys^7]CSF114 (**2**): HPLC-MS experiment was performed using Alliance Chromatography model 2695 (*Waters*) with a Phenomenex Kinetex C18 column (2.6 µm, 3.0 × 100 mm) working at 0.6 mL/min, coupled to a single quadrupole ESI-MS (*Micromass ZQ*) at 6 mL/min of: (A) 0.1% TFA in H_2O *MilliQ* and (B) 0.1% TFA in 84% MeCN/H_2O, λ=254 nm, gradient: 10-90% B in 5 min, injection volume: 10 µL.

For the deoxyfructosylated peptides **2** and **18** in the hydrolysate of Human Serum Albumin or Bovine Serum Albumin: HPLC-MS/MS experiment was performed using Aeris C18 column (3.6 µm, 2.1 × 100 mm) working at 0.2 mL/min, coupled to IT-TOF ESI-MS (*Shimadzu*) at 6 mL/min of: (A) 0.1% HCOOH in H_2O and (B) 0.1% HCOOH in MeCN, gradient: 0-55% B in 40 min, injection volume: 1-10 µL.

UV-Vis analysis

Plate reader: Tecan infinite M200 Pro, Tecan Group Ltd, Männedorf, Switzerland, Cuvette measurement mode with blanking.

Preparation of the calibration curves of 4-carboxyphenylboronic acid

6.64 mg (4×10^{-5} mol) of 4-carboxyphenylboronic acid (Sigma Aldrich, M=165.94 g/mol) was dissolved in 10 ml of H_2O/MeCN 1:1 (*v/v*) to the concentration of 4 mM. The sequence of calibration samples was prepared by serial dilution of the initial sample. H_2O/MeCN 1:1 (*v/v*) mixture was used as blank sample. The samples were measured at wavelength 236 nm (**Fig. S4**).

Preparation of the calibration curve of Fmoc-Lys(Dabcyl)-OH (**I**) *in capturing mixture ammonium bicarbonate buffer*

3.09 mg (5×10^{-6} mol) of Fmoc-Lys(Dabcyl)-OH (M=619.71 g/mol) was dissolved in 5 ml of ammonium bicarbonate buffer in H_2O/MeCN 1:1 (*v/v*) and then diluted 10 times to 0.1 mM concentration. The sequence of calibration samples was prepared by serial dilution of the initial sample. Ammonium bicarbonate buffer H_2O/MeCN 1:1 (*v/v*) was used as blank sample (**Fig. S11**).

Preparation of the calibration curve of Fmoc-K(Dabcyl)-OH (**I**) *in the cleavage mixture (0.1% HCOOH)*

3.09 mg (5 × 10^{-6} mol) of Fmoc-Lys(Dabcyl)-OH (M=619.71 g/mol) was dissolved in 5 ml 0.1% HCOOH in H_2O/MeCN 1:1 (*v/v*) and then diluted 10 times to 0.1 mM concentration. The sequence of calibration samples was prepared by serial dilution of the initial sample. The solutiion 0.1% HCOOH in H_2O/MeCN 1:1 (*v/v*) was used as blank sample (**Fig. S12**).

Enzymatic hydrolysis of HSA

Enzymatic hydrolysis was performed according the protocol previously reported [*Waliczek M, Bąchor R, Kijewska M, Gąszczyk D, Panek-Laszczyńska K, Konieczny A, Dąbrowska K, Witkiewicz W, Marek-Bukowiec K, Tracz J, Łuczak M, Szewczuk Z, Stefanowicz P. Isobaric duplex based on a combination of 16O/18O enzymatic exchange and labeling with pyrylium salts. Anal Chim Acta 2019, 1048, 96-104*] Briefly, 1 mg HSA was dissolved in 200 µl of 50 mM ammonium bicarbonate buffer, complemented with 5 µl of 200 mM dithiothreitol in 50 mM ammonium bicarbonate buffer and incubated for 45 min. at 60°C. Afterwards, the sample was cooled to RT, before 4 µl of 1M iodoacetamide in 50 mM ammonium bicarbonate buffer was added, and alkylation of free sulfhydryls was performed during 1 h in darkness at RT. To decompose unreacted iodoacetamide 20 µl of DTT was added. Then 50 µl of trypsin stock solution (1 mg in 1000 µl in water) was added to reach the enzyme: substrate mass ratio of 1:20 and incubated for 12 h at 37°C. Digestion was terminated by the addition of 10 µl of formic acid. The resulting digest was lyophilized and used for MS experiments.

The PhB-Lys(PhB)-ChemMatrix-Rink Resin Is Specific For Deoxyfructosylated Peptides

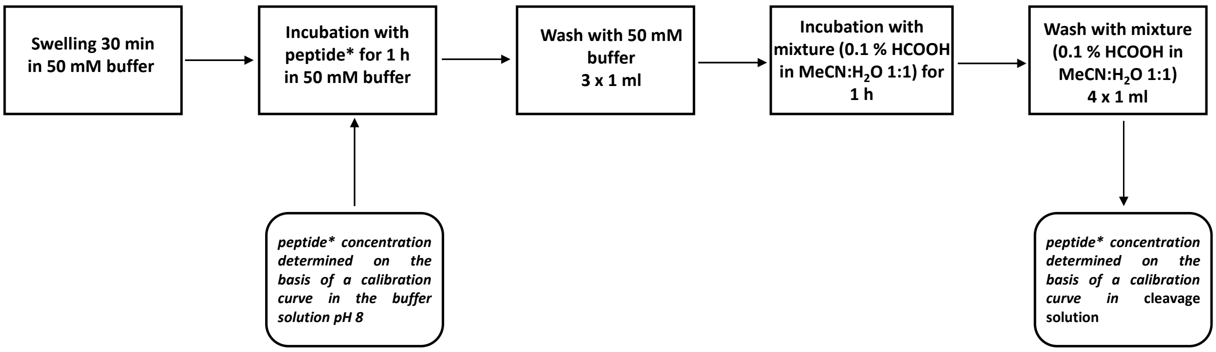

Scheme S1. Procedure of capturing and cleavage of deoxyfructosylated peptides by the Resin Peptide*: H-K(Dabcyl)AK(1-DeoxyFru)AF-NH_2 (**1**)

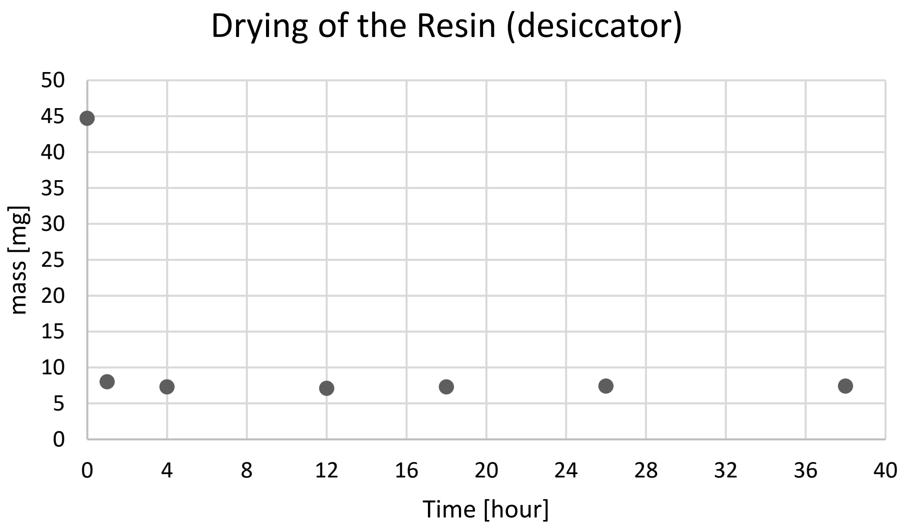

Fig. S14 Plot reporting the drying time of the ChemMatrix® Rink resin functionalised with the linker

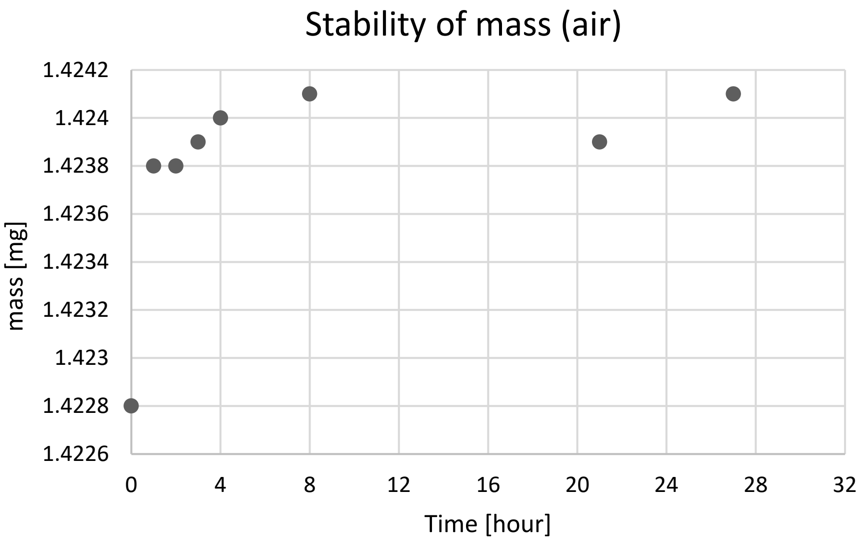

Fig. S15 Plot reporting the mass stability of ChemMatrix® Rink resin functionalised with the linker as a function of time

The PhB-Lys(PhB)-ChemMatrix® Rink Resin Is Specific for Deoxyfructosylated Peptides and not for Differently Glycosylated Peptides

Preparation of the calibration curve of the glycosylated peptides (**2-8**)

Calibration curve to determine the amount of the peptide CSF114 (**8**) linked to the resin was adjusted as follows: five dilutions of pure CSF114 were prepared (4:1 – 0.001535 M, 2:1 – 0.0007675 M, 1:1 – 0.00038373 M, 1:10 – 0.0000384 M and 1:50 – 0.00000767 M, where 1:1 means 1 mg/mL solutions in H_2O). Each sample was measured threefold on LC-MS instrument Alliance Chromatography with Micromass ZQ (Waters); column: Kinetex C18 2.6 μm, 3 x 100 mm (Phenomenex); eluents: A: 0.1% TFA in H_2O, B: 0.1% TFA in 84 % ACN; flow: 0,6 mL/min; gradient 10-90% B in 5 min. Obtained peak areas were then integrated, media from three independent values for each point were taken and the plots of calibration curves were prepared.

Calibration curves for the other peptides were performed according to the procedure described above.

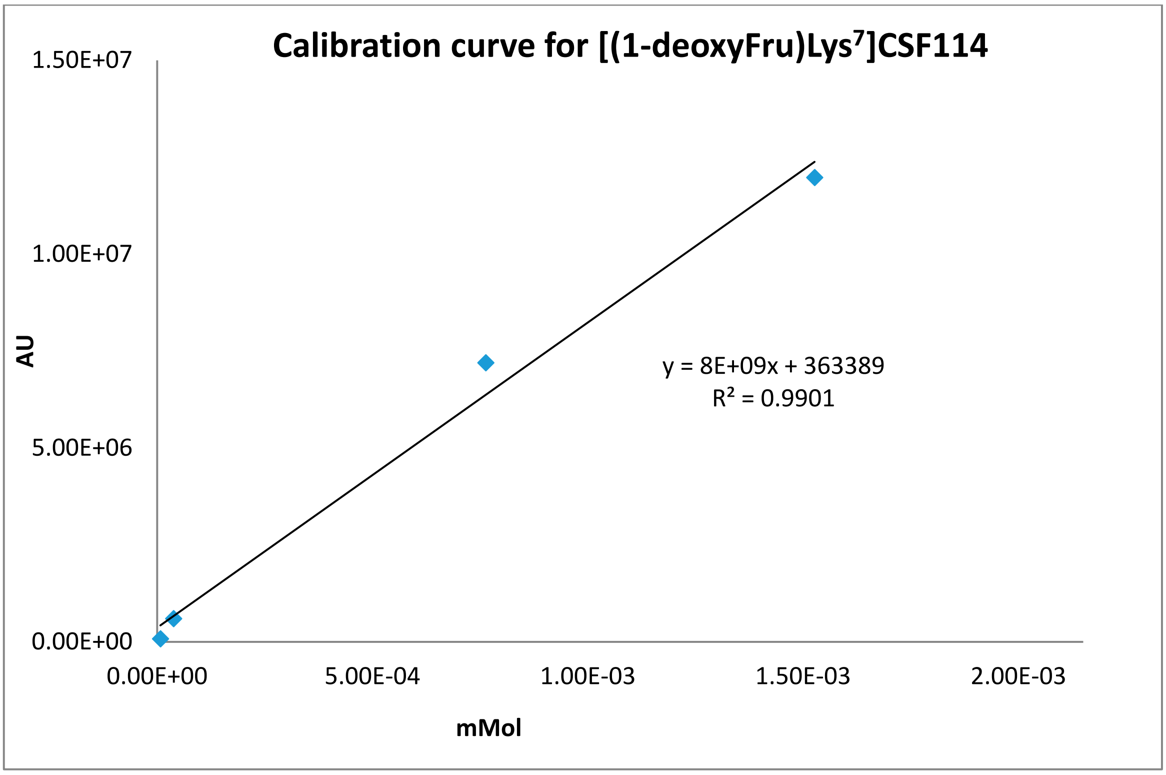

Fig. S16 Calibration curve of [(1-DeoxyFru)Lys^7]CSF114 (**2**)

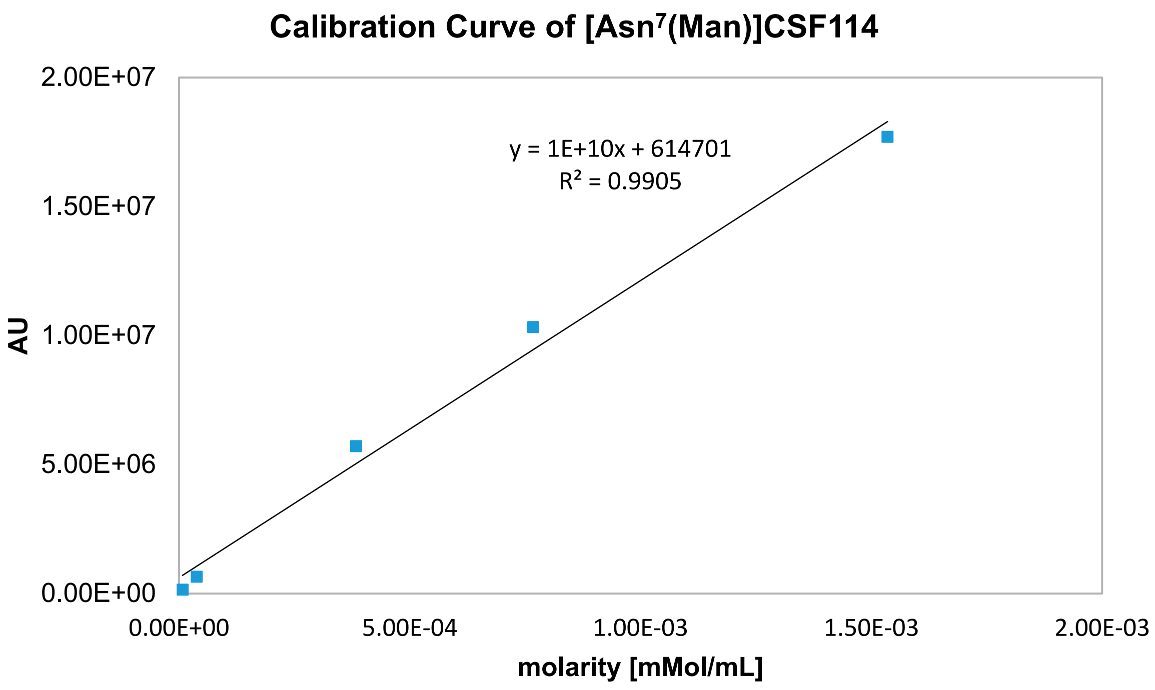

Fig. S17 Calibration curve of [Asn^7(Man)]CSF114 (**3**)

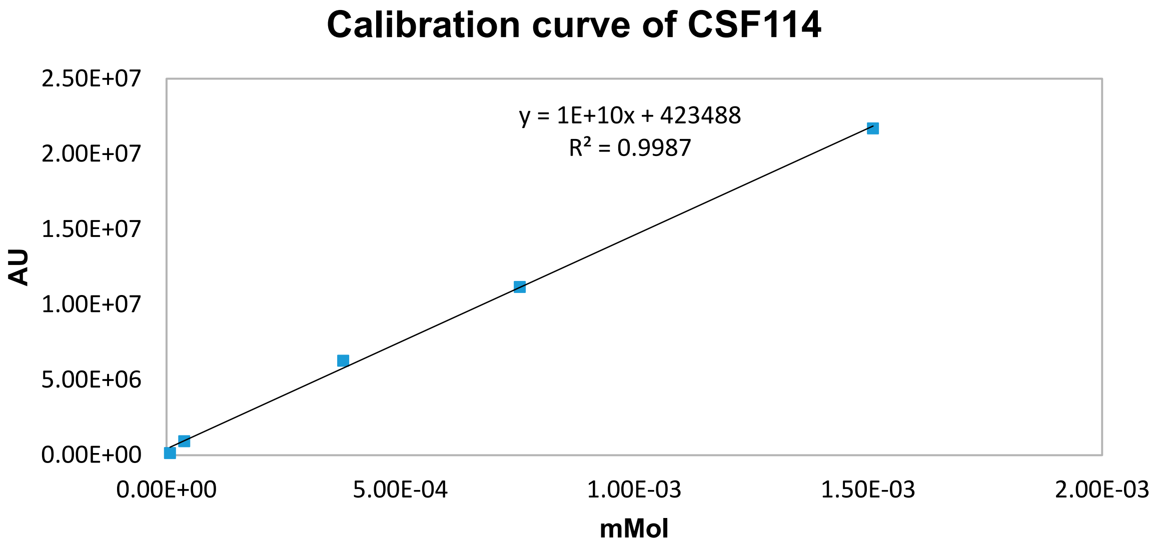

Fig. S18 Calibration curve of CSF114 (**8**)

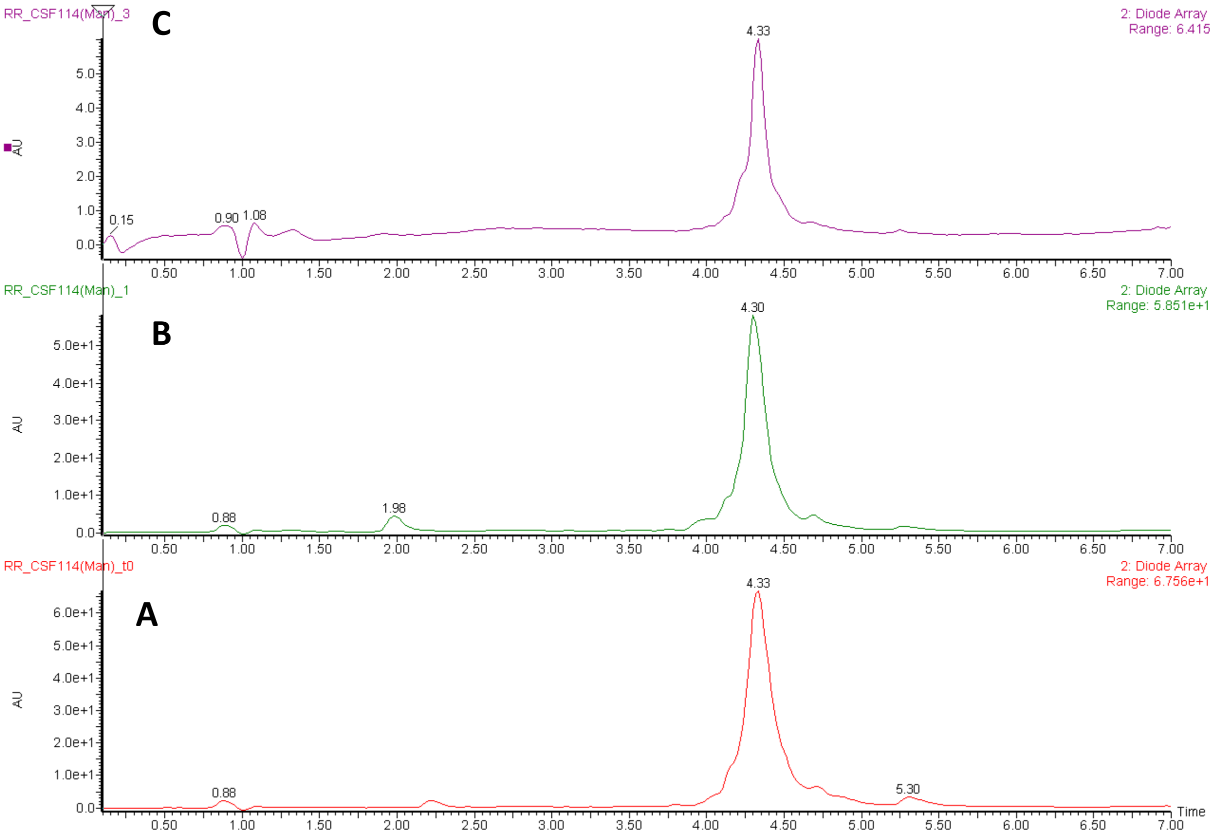

Fig. S19 Chromatograms of the peptide [Asn^7(Man)]CSF114 (**3**). Panel (**A**): t = 0. Panel (**B**): fraction not captured by the resin. Panel (**C**): fraction after capturing procedure

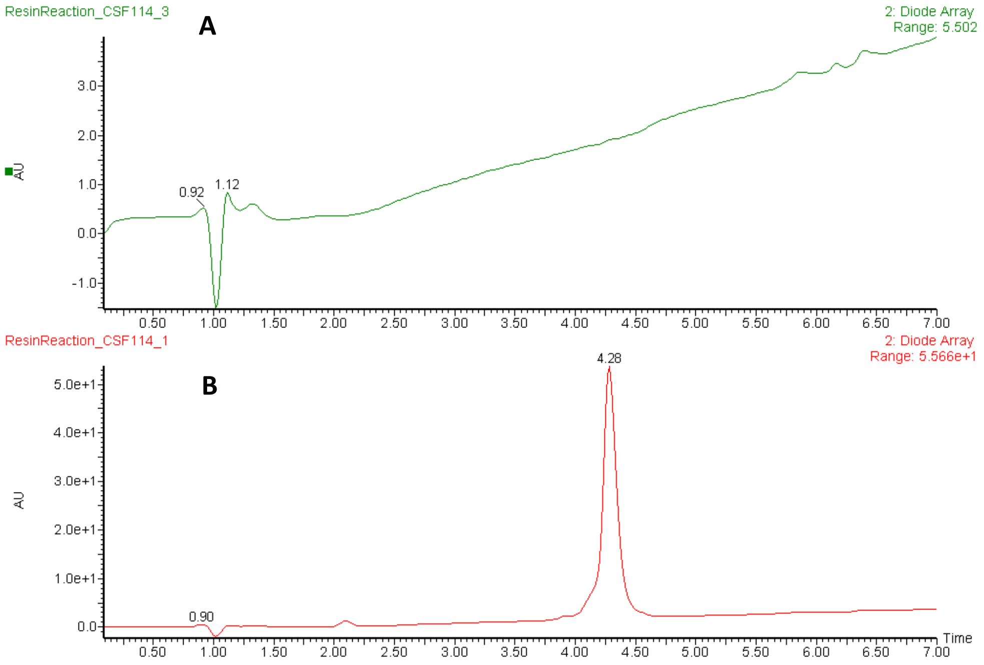

Fig. S20 Chromatograms of the peptide CSF114 (**8**). Panel (**A**): fraction after capturing procedure. Panel (**B**): fraction not captured by the resin

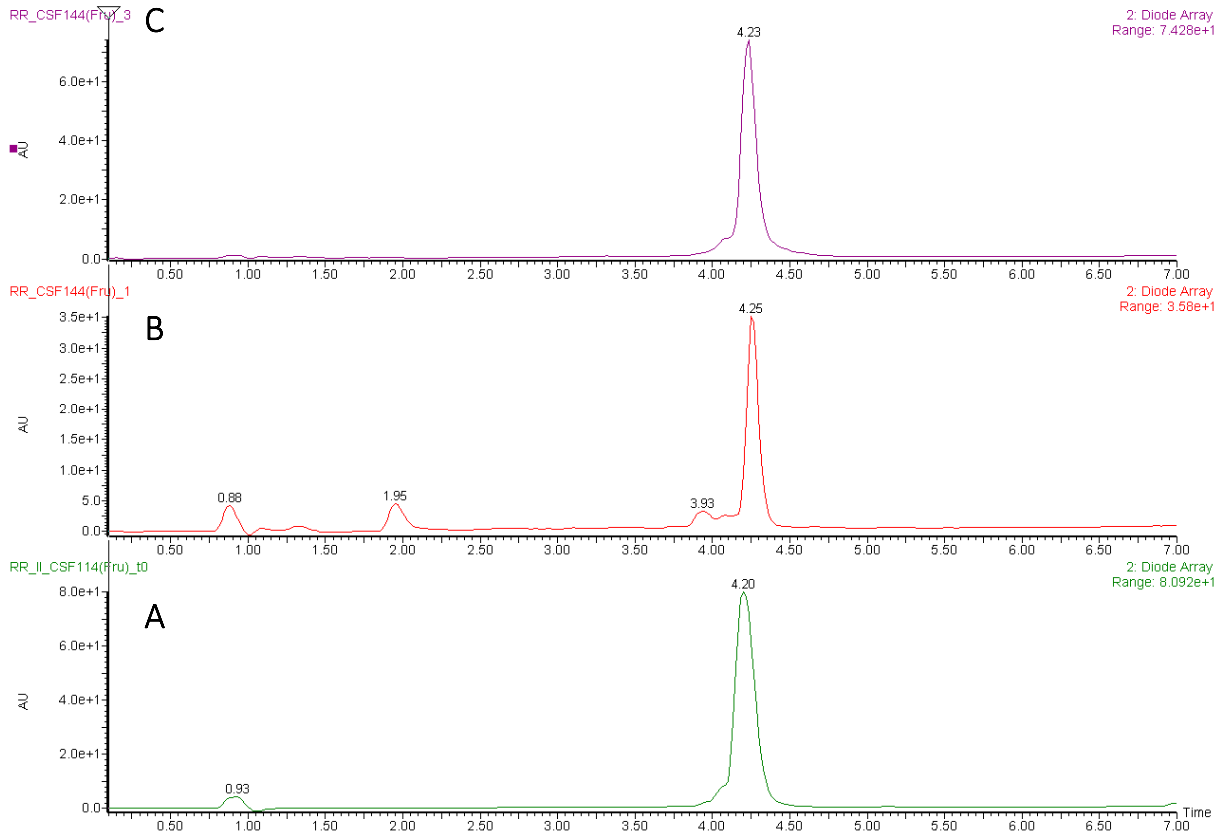

Fig. S21 Chromatograms of the peptide [(1-DeoxyFru)Lys^{7}]CSF114 (**2**). Panel (**A**): t = 0. Panel (**B**): fraction not captured by the resin. Panel (**C**): fraction after capturing procedure

The PhB-Lys(PhB)-ChemMatrix® Rink Resin Is Specific For Deoxyfructosylated Peptides and Not for Unrelated Non-Glycosylated Peptides

Table S2. Synthetic peptides used as a matrix for capturing of [(1-deoxyFru)Lys^7]CSF114 (**2**)

N	Peptide	ESI-MS (m/z) (Exact Mass $_{calcd}$)[a] found[b]	HPLC (t_R, min)[c]
**2**	[(1-DeoxyFru)K^7]TPRVERGHSVFLAPYGWMVK	(2620) 874.8 (3+)	4.27
**9**	Ac-GKNAT	(530) 531(1+)	1.10
**10**	Ac-MSKVVNPTQK-NH$_2$	(1312) 1313 (1+)	3.30
**11**	LSETTI	(662) 663 (1+)	3.35
**12**	DQDAEQAPEYRGRTELLKET	(2346.2) 1175 (2+)	3.42
**13**	EKEK	(802) 803 (1+)	3.45
**14**	REKLVVRRGQPFWLTLHFEGR	(2625) 1313 (2+)	4.20
**7**	(PO$_3$H$_2$)S^{7}TPRVERGHSVFLAPYGWMVK	(2489.9) 1249 (2+)	4.23
**15**	GQFRVIGPGYPIRALVGDEAELPCRISPGKNATG	(3535.8) 1770 (2+)	4.27
**16**	ENPVVHFFKNIVTPRTP	(2036.1) 1019 (2+)	4.67
**17**	Pam-ENPVVHFFKNIVTPRT	(2232.4) 1117 (2+)	6.80

[a]ESI-MS: detected as [a][M+H]$^+$; [b c] [M+H]$^+$[M+3H]$^{3+}$. Solvent system A: 0.1% TFA in H_2O, B: 0.1% TFA in CH_3CN. Analytical HPLC gradients at 0.6 mL min^{-1}: [c] 10-90% B in 5 min.

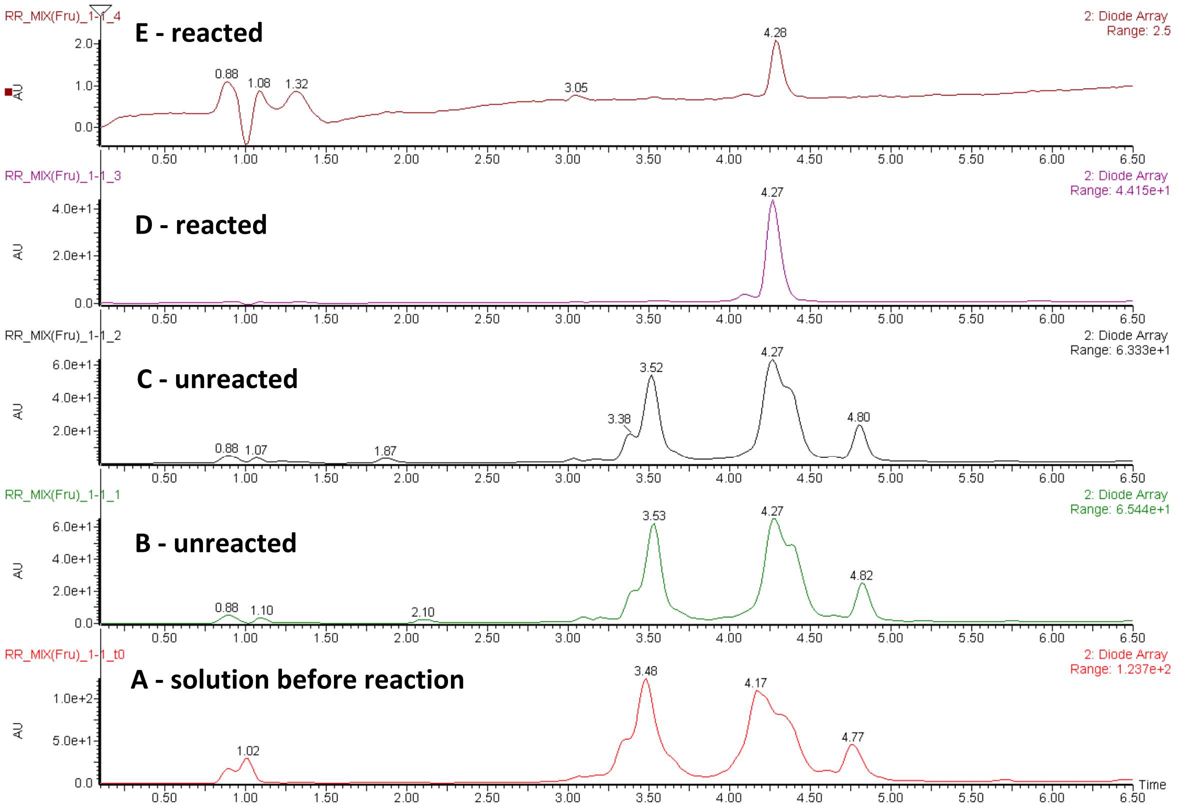

Fig. S22 Chromatograms of equimolar mixture of the non-glycosylated peptides and [(1-DeoxyFru)Lys^{7}]CSF114. Panel (**A**): HPLC of the solution before capturing procedure. Panel (**B**): HPLC of the fraction not captured by the resin. Panel (**C**): HPLC of the fraction washed with the buffer solution after the capturing procedure. Panel (**D**): HPLC of the fraction captured by the resin. Panel (**E**): HPLC of the fraction washed with the cleavage mixture after reaction with the resin

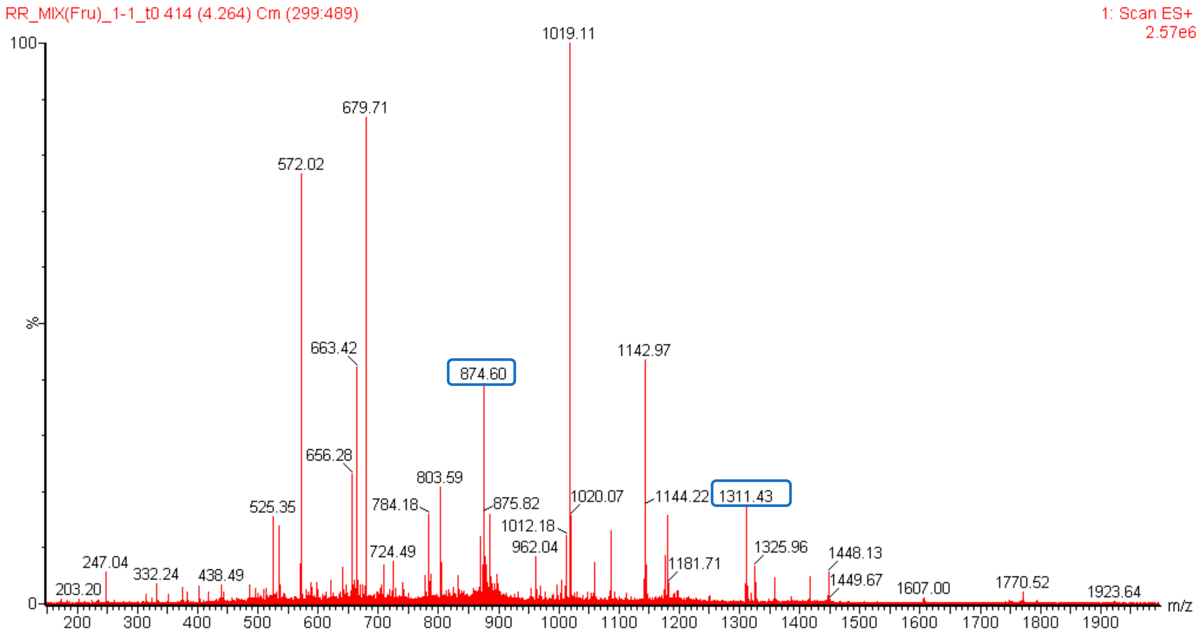

Fig. S23 ESI-MS spectrum of an equimolar mixture of the non-modified peptides and [(1-DeoxyFru)Lys^{7}]CSF114 (**2**). Solution before the reaction.

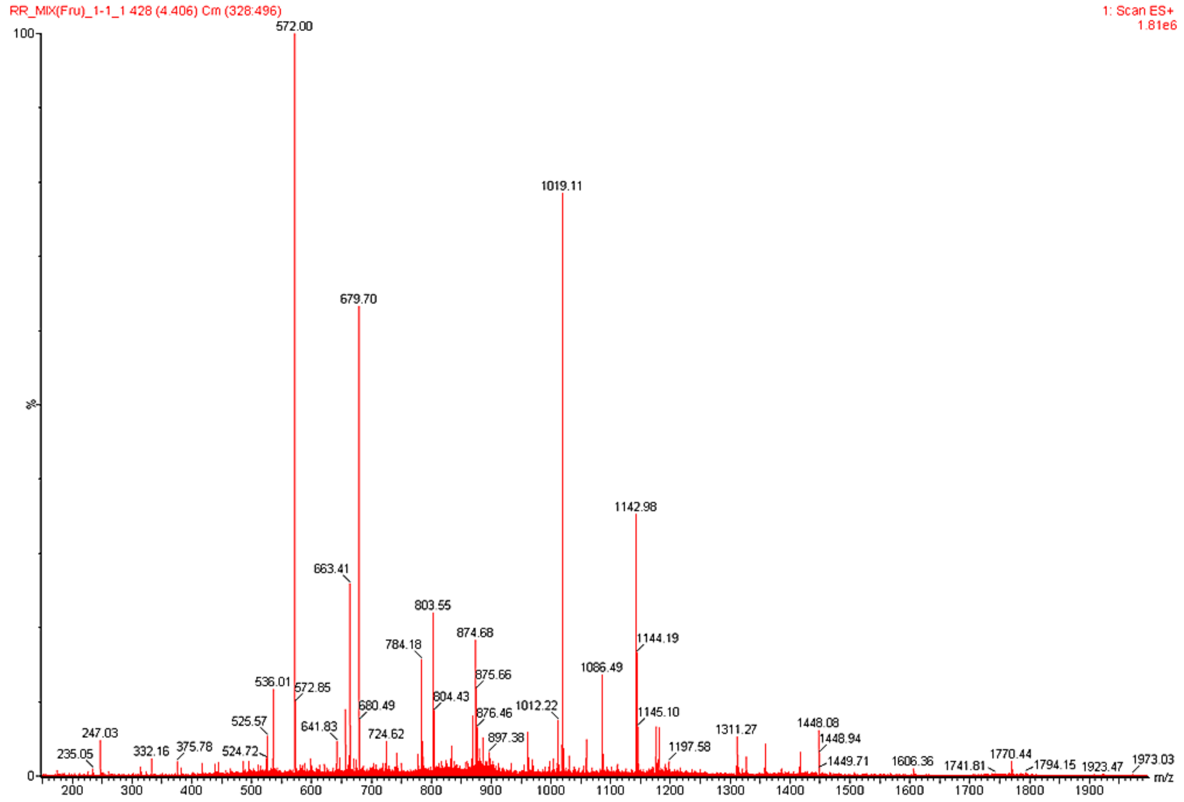

Fig. S24 ESI-MS spectrum of an equimolar mixture of the non-modified peptides and [(1-DeoxyFru)Lys^{7}]CSF114 (**2**). Unreacted fraction.

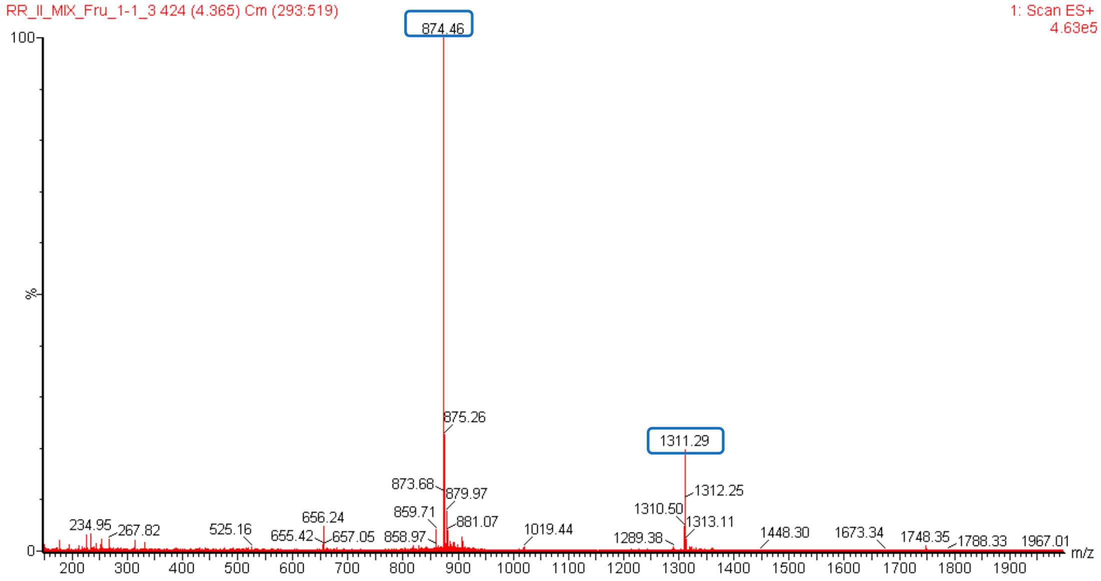

Fig. S25 ESI-MS spectrum of an equimolar mixture of the non-modified peptides and [(1-DeoxyFru)Lys^{7}]CSF114 (2). Captured fraction.

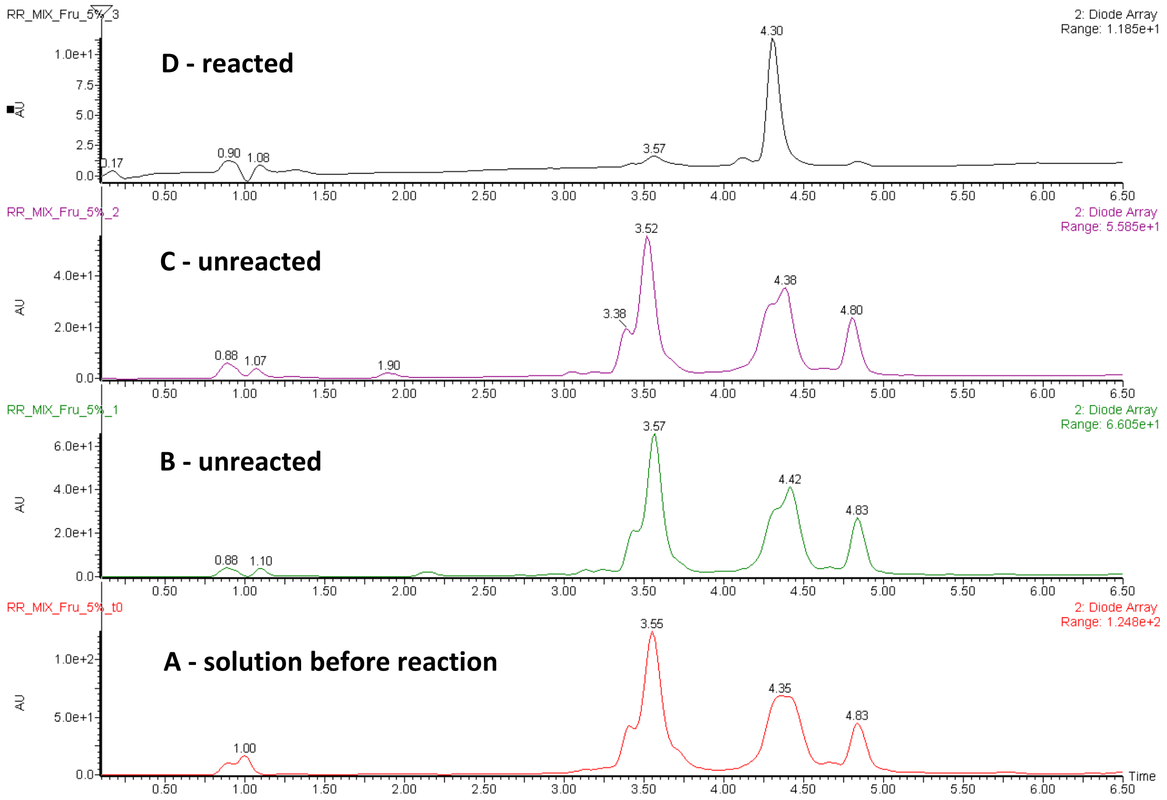

Fig. S26 Chromatograms of the mixture of the non-glycosylated peptides and 5% w/w (10.5 nmol/mL) [(1-DeoxyFru)Lys^{7}]CSF114 (**2**). Panel (**A**): HPLC of the solution before capturing procedure. Panel (**B**): HPLC of the fraction which was not captured by the resin. Panel (**C**): HPLC of the fraction washed with the buffer solution after the capturing procedure. Panel (**D**): HPLC of the fraction captured by the resin.

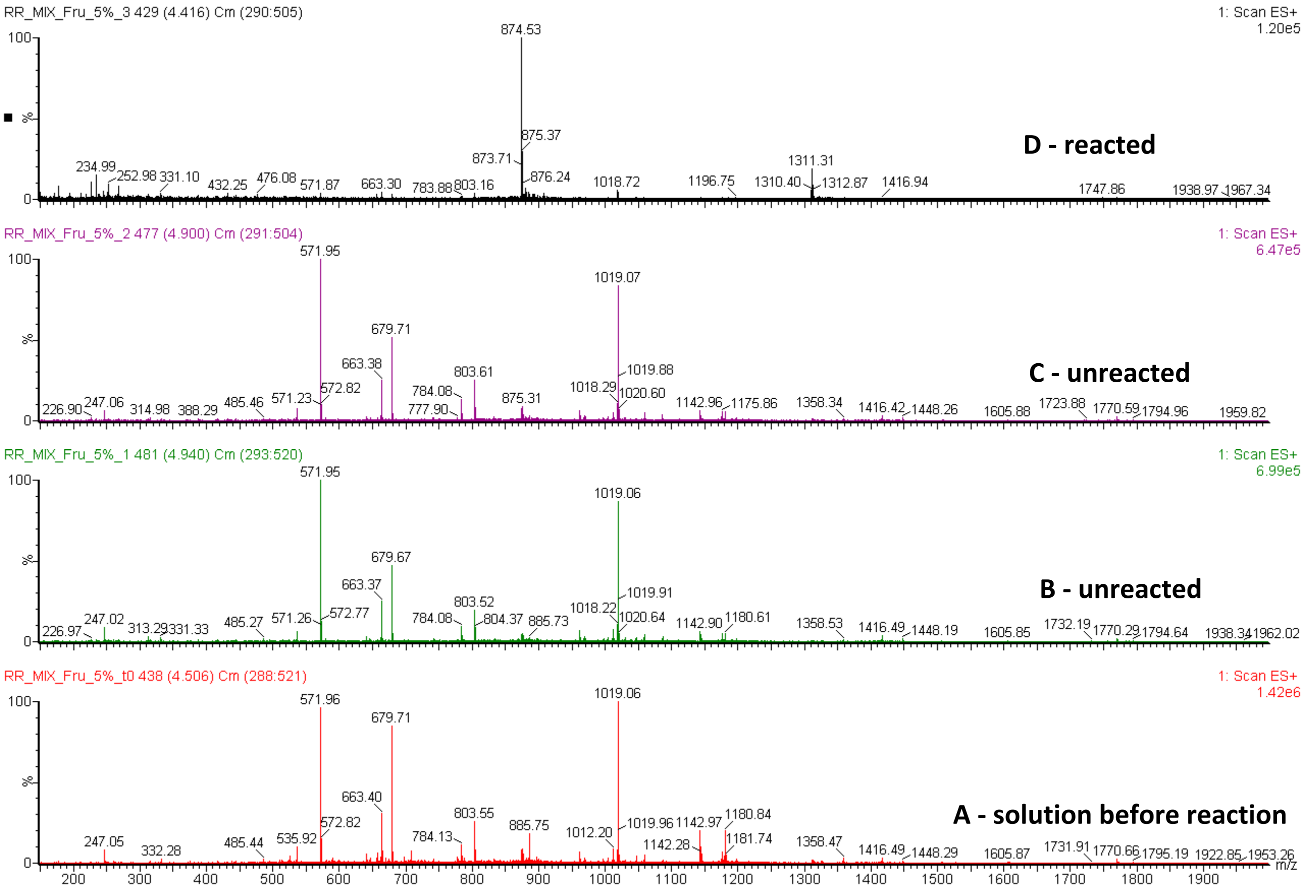

Fig. S27 ESI-MS of the mixture of the non-glycosylated peptides and 5% *w/w* (10.5 nmol/mL) [(1-DeoxyFru)Lys^{7}]CSF114 (**2**). Panel (**A**): ESI-MS of the solution before capturing procedure. Panel (**B**): ESI-MS of the fraction which was not captured by the resin. Panel (**C**): ESI-MS of the fraction washed with the buffer after the capturing procedure. Panel (**D**): ESI-MS of the fraction captured by the resin.

Assay to Investigate the Specificity Of CMRR for Deoxyfructosylated Peptides in Hydrolysate of Human Serum Albumin and Bovine Serum Albumin

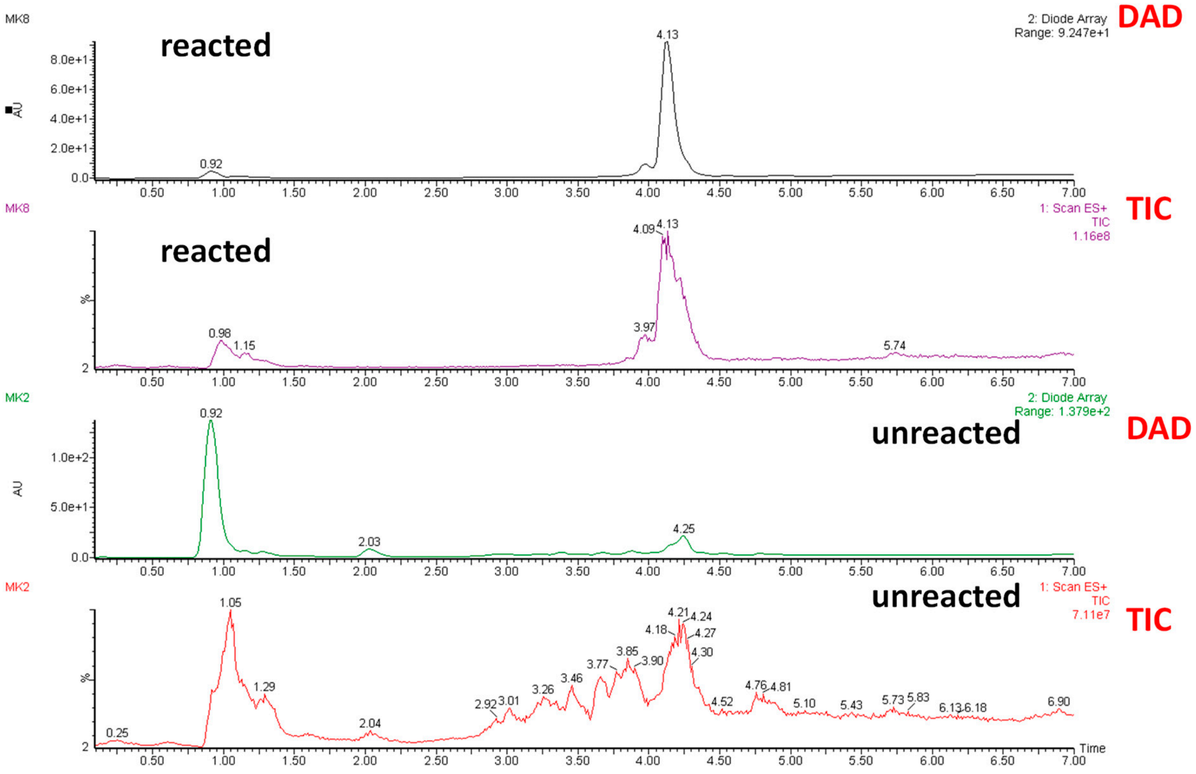

Fig. S28 Chromatograms of the hydrolysate of HSA with 1.4 μmol/mL of [(1-DeoxyFru)Lys[7]]CSF114 (**2**).

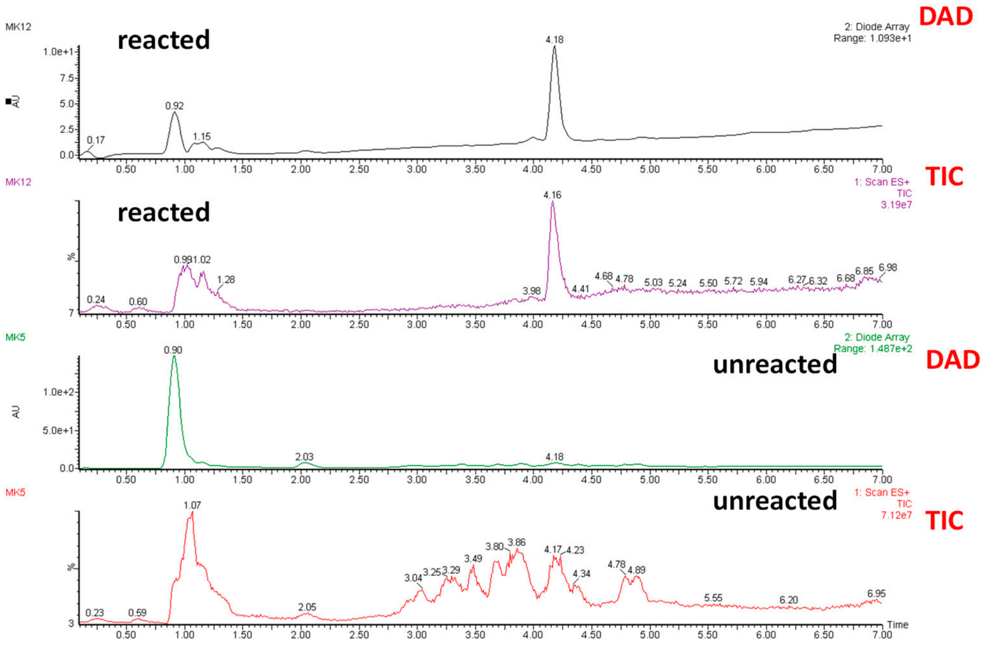

Fig. S29 Chromatograms of the hydrolysate of HSA with 5% *w/w* (10.5 nmol/mL) and [(1-DeoxyFru)Lys7]CSF114 (**2**).

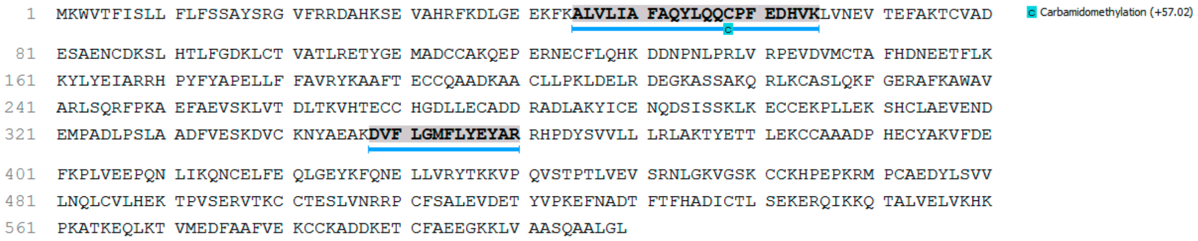

Fig. S30 Protein coverage based on LC-MS analysis of 5% *w/w* (10.5 nmol/mL) of [(1-DeoxyFru)Lys[7]]CSF114 (**2**) in the hydrolysate of HSA

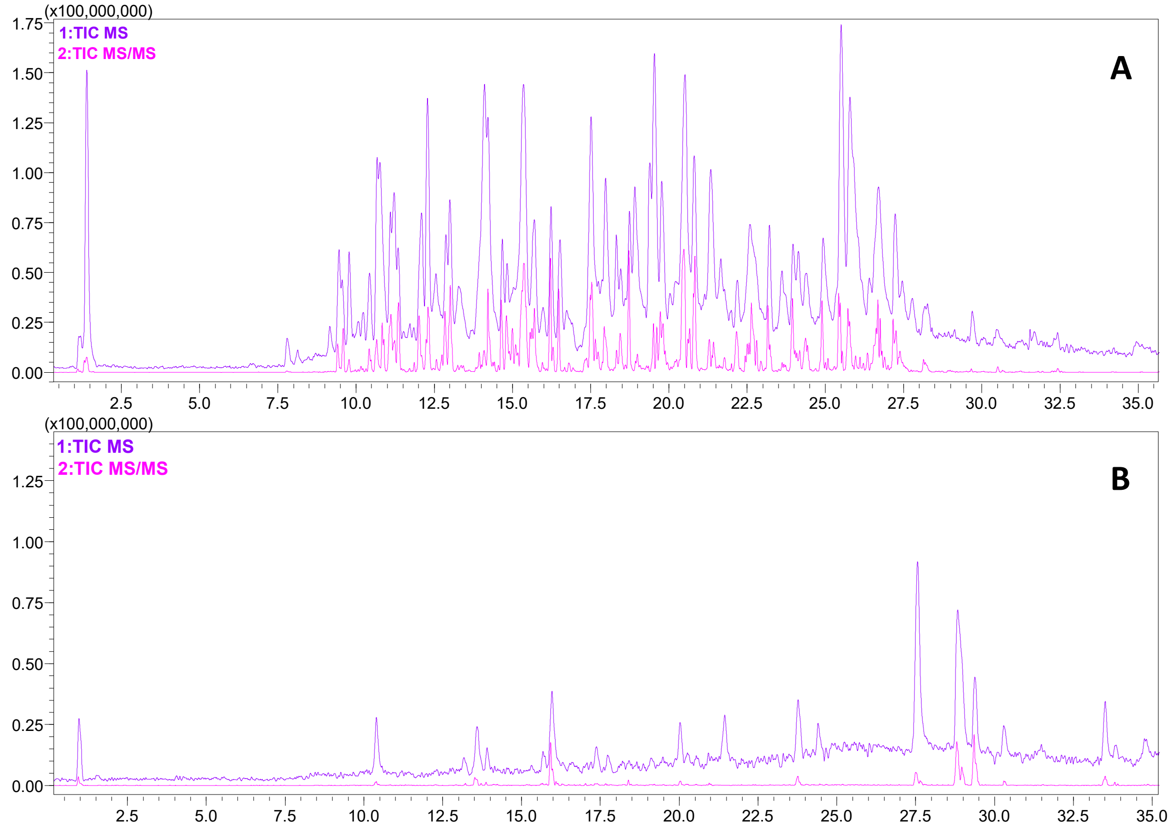

Fig. S31 LC-MS analysis of the resin capturing reaction without the peptide [(1-DeoxyFru)Lys[7]]CSF114 (**2**) in the hydrolysate of HSA (Blank). Panel (**A**): before the capturing reaction with the resin. Panel (**B**): after the capturing reaction with the resin

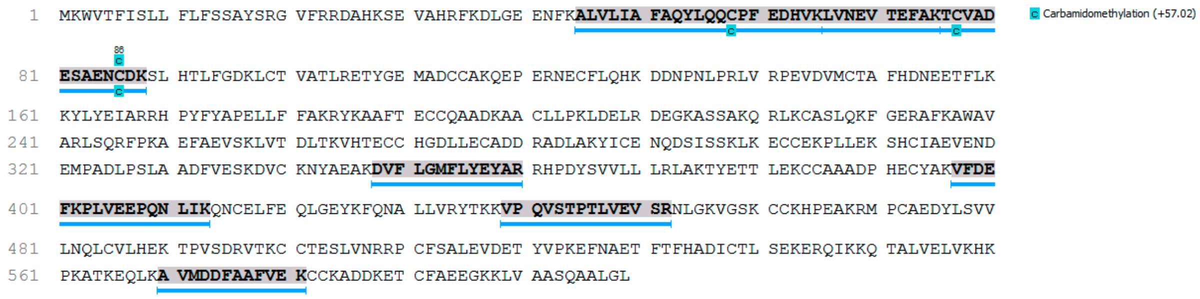

Fig. S32 Protein coverage based on LC-MS analysis of the capturing resin reaction without the peptide [(1-DeoxyFru)Lys^{7}]CSF114 (**2**) in the hydrolysate of HSA (blank)

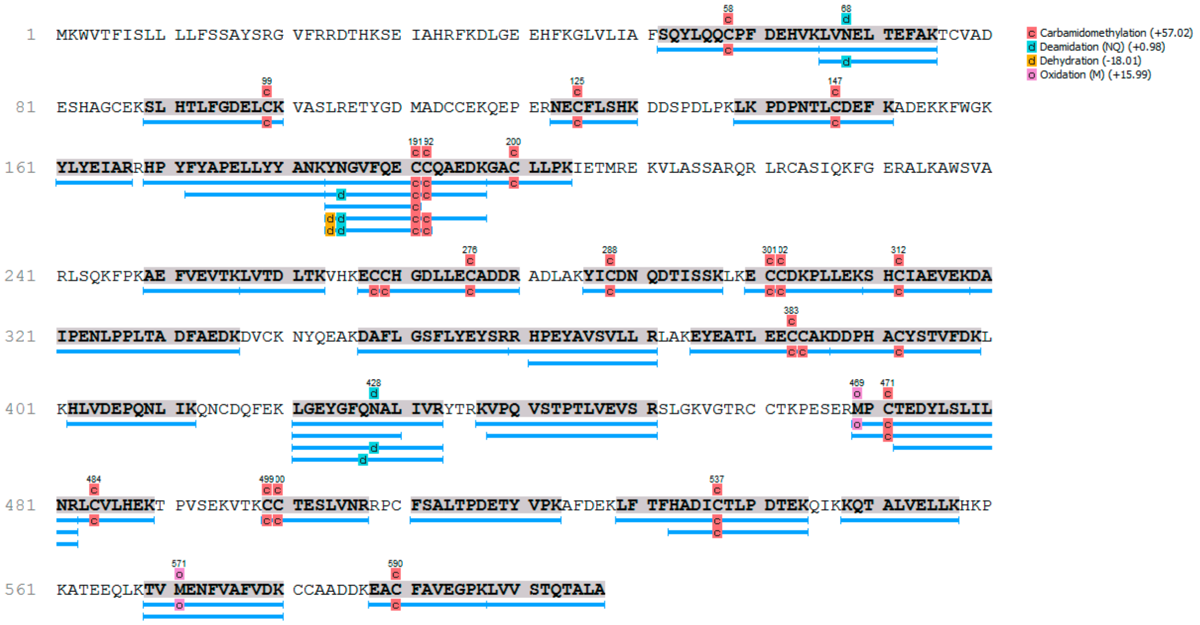

Fig. S33 Protein coverage based on LC-MS analysis of 500 pmol hydrolysate of BSA (BioLabs) with 300 pmol of the deoxyfructosylated peptide DTEK(1-DeoxyFru)QIKKQT

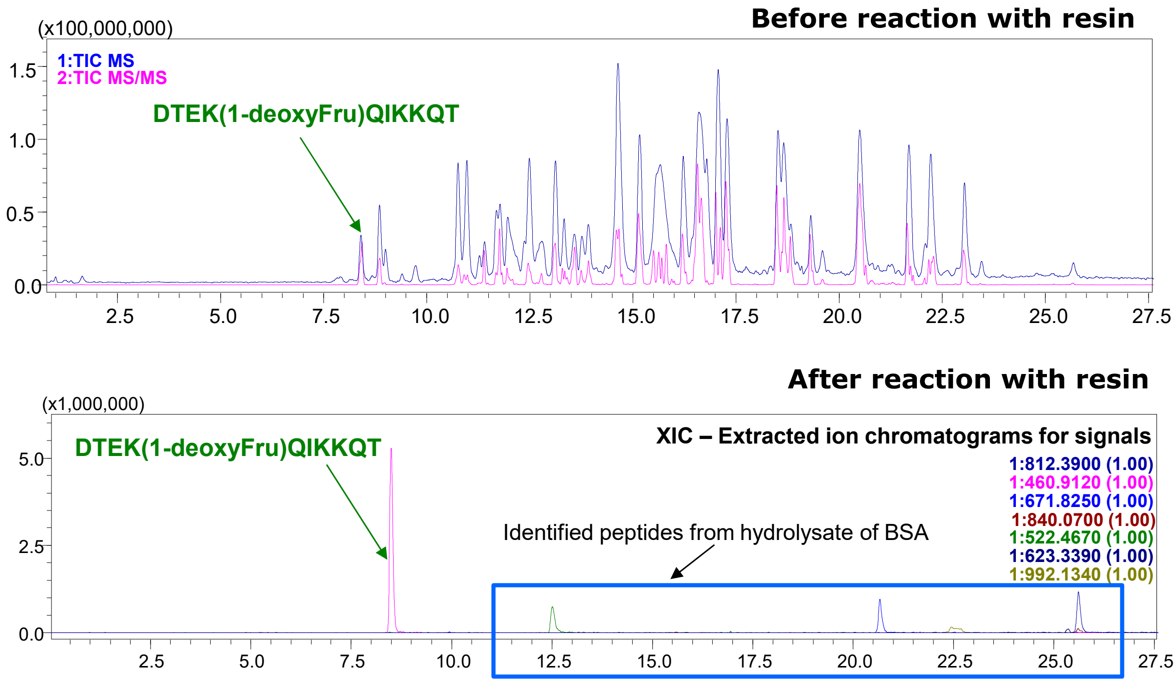

Fig. S34 LC-MS/MS analysis of 500 pmol hydrolysate of BSA (BioLabs) with 300 pmol of the deoxyfructosylated peptide DTEK(1-DeoxyFru)QIKKQT before the capturing reaction with the resin and after the capturing reaction with the resin

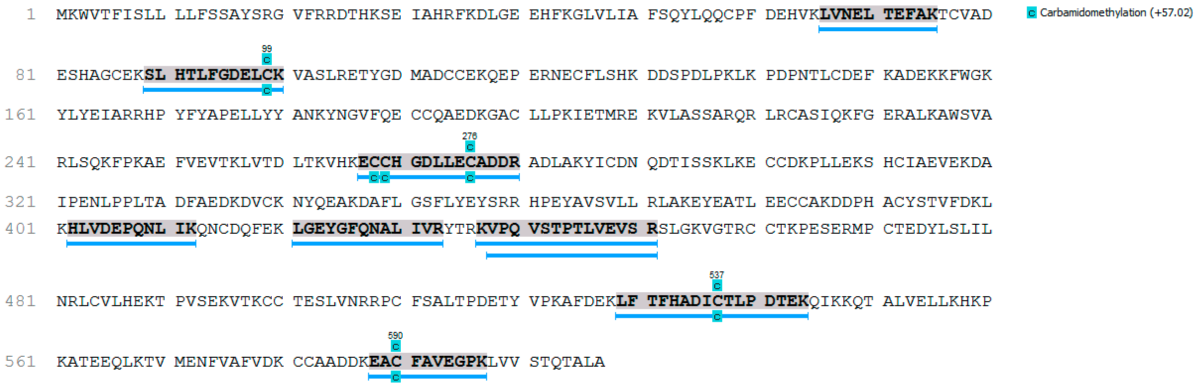

Fig. S35 Protein coverage based on HPLC-MS/MS analysis of the capturing resin reaction with 500 pmol hydrolysate of BSA (BioLabs) with 300 pmol of deoxyfructosylated peptide DTEK(1-DeoxyFru)QIKKQT
